# Long-Term Biocide Efficacy and Its Effect on a Souring Microbial Community

**DOI:** 10.1128/AEM.00842-21

**Published:** 2021-08-11

**Authors:** Xiang Shi, Daiane A. F. Oliveira, Lea Holsten, Katrin Steinhauer, Julia R. de Rezende

**Affiliations:** a The Lyell Centre, Heriot-Watt University, Edinburgh, United Kingdom; b Department of Oceanography, Federal University of Bahia, Salvador, Brazil; c Schülke & Mayr GmbH, Norderstedt, Germany; Shanghai Jiao Tong University

**Keywords:** reservoir souring, biocide, oilfield microbiology, sulfate reduction, microbial control, glutaraldehyde

## Abstract

Reservoir souring, which is the production of H_2_S mainly by sulfate-reducing microorganisms (SRM) in oil reservoirs, has been a long-standing issue for the oil industry. While biocides have been frequently applied to control biogenic souring, the effects of biocide treatment are usually temporary, and biocides eventually fail. The reasons for biocide failure and the long-term response of the microbial community remain poorly understood. In this study, one-time biocide treatments with glutaraldehyde (GA) and an aldehyde-releasing biocide (ARB) at low (100 ppm) and high (750 ppm) doses were individually applied to a complex SRM community, followed by 1 year of monitoring of the chemical responses and the microbial community succession. The chemical results showed that souring control failed after 7 days at a dose of 100 ppm regardless of the biocide type and lasting souring control for the entire 1-year period was achieved only with ARB at 750 ppm. Microbial community analyses suggested that the high-dose biocide treatments resulted in 1 order of magnitude lower average total microbial abundance and average SRM abundance, compared to the low-dose treatments. The recurrence of souring was associated with reduction of alpha diversity and with long-term microbial community structure changes; therefore, monitoring changes in microbial community metrics may provide early warnings of the failure of a biocide-based souring control program in the field. Furthermore, spore-forming sulfate reducers (*Desulfotomaculum* and *Desulfurispora*) were enriched and became dominant in both GA-treated groups, which could cause challenges for the design of long-lasting remedial souring control strategies.

**IMPORTANCE** Reservoir souring is a problem for the oil and gas industry, because H_2_S corrodes the steel infrastructure, downgrades oil quality, and poses substantial risks to field personnel and the environment. Biocides have been widely applied to remedy souring, but the long-term performance of biocide treatments is hard to predict or to optimize due to limited understanding of the microbial ecology affected by biocide treatment. This study investigates the long-term biocide performance and associated changes in the abundance, diversity, and structure of the souring microbial community, thus advancing the knowledge toward a deeper understanding of the microbial ecology of biocide-treated systems and contributing to the improvement of current biocide-based souring control practices. The study showcases the potential application of incorporating microbial community analyses to forecast souring, and it highlights the long-term consequences of biocide treatment in the microbial communities, with relevance to both operators and regulators.

## INTRODUCTION

Oil reservoirs are deep subsurface ecosystems that are known to host large and diverse populations of indigenous and exogenous microorganisms, including sulfate reducers, nitrate reducers, iron reducers, and fermenters (1). The metabolic activity of those microorganisms and their interactions with the subsurface environment have been known to cause negative issues for the oil and gas industry, and reservoir souring is one such issue that attracts wide attention (2). Reservoir souring refers to the increase of hydrogen sulfide (H_2_S) in the produced fluid over the lifetime of a field. It is usually associated with the implementation of enhanced oil recovery techniques involving injection of large volumes of water into the reservoir (3). Reservoir souring can occur abiotically through various mechanisms (4–6), but it is most commonly attributed to the metabolic activity of sulfate-reducing microorganisms (SRM) taking place in oil reservoirs (7, 8). H_2_S corrodes the production facilities, increases the environmental risk of leaks and spills, reduces oil quality, and poses risks to the health and safety of field personnel due to its corrosivity and toxicity. Therefore, effective strategies to control souring have been in demand for decades (8, 9).

Biocides have been routinely applied in the industry to control a wide range of microbial problems, such as microbiologically influenced corrosion, biofouling, and reservoir souring. The existing studies related to the use of biocides to control reservoir souring are mainly efficacy tests that focus on determining the effective doses of biocides. Such tests are typically performed using selected biocides, with laddered concentrations applied on pure cultures of SRM, and tests are often conducted on the order of days or weeks. A study performed by Yin and colleagues provides a good example demonstrating the common workflow of such tests (10). These tests are useful in providing a standardized reference to facilitate determination of the minimum inhibitory concentration (11), but they face inescapable limitations. First, when commercial strains are used for such tests, the results may not reflect the complex interactions among the members of a natural microbial community. More critically, in a natural environment over the long term, a part of the microbial community is expected to survive the biocide, leading to microbial community changes and eventually resulting in souring control failure (11). The conventional tests aimed at determining biocide doses reveal only the short-term effects of a biocide treatment and do not investigate the souring recovery process after the failure of the treatment over the longer term.

From a microbial ecology point of view, treating a microbial community with biocides essentially introduces environmental disturbances to the community. In response to the disturbances, the microbial community members may die or change in their relative abundances (12), leading to changes in some ecological properties of the microbial community. A batch study by Campa and colleagues investigated the responses of surface water microbial communities to glutaraldehyde (GA) treatment for 56 days and confirmed that biocides may affect the ecology of the surface water microbial community (13). The study amended enrichments of different types of stream water samples with 100 ppm GA. The biocide treatment steadily reduced the diversity of the microbial community. The treatment resulted in pronounced differences in the microbial community structures between the treated and corresponding blank control groups toward the end of the experiment. Degradation of GA by the microbial community was also observed during the process. Campa and colleagues also investigated the microbial community response to 2,2-dibromo-3-nitrilopropionamide (DBNPA) following a similar workflow (14). In that study, the microbial response to DBNPA was distinct, compared with the response to GA, and a different microbial community structure resulted from the DBNPA treatment (14). To our knowledge, however, no studies have yet explored the relationships between biocide performance and the ecological properties of a souring microbial community during biocide treatment and after biocide failure, particularly how the ecological property changes could reflect the failure of biocides in souring control.

In the present study, we applied one-time biocide treatments to a complex SRM community in a souring scenario. For 1 year, we monitored the concentration of sulfide and carbon sources and tracked the microbial community succession. We assessed the duration of effective souring control and evaluated the souring recovery process after biocide failure. We also investigated the changes in key microbial ecology properties of the souring microbial community facing the biocide treatments that could be related to the long-term biocide failure. The key ecological properties investigated were the abundance, diversity, and structure of the microbial community. Particularly, we used alpha diversity metrics (the Shannon index and the Berger-Parker index) and beta diversity metrics (weighted UniFrac distance matrix and the relative abundance profile of the community) to quantitatively represent the diversity and structure of the community. We were able to identify implications based on these ecological properties that were associated with the success or failure of the biocide treatment. The findings contribute to guiding the field practice to recognize early signs of souring control failure. The findings also provide insights to improve the risk assessment of the biocide program by taking into consideration the long-term biocide performance and the associated microbial community responses. Collectively, this study demonstrates the importance and potential benefits of understanding the ecological properties of biocide-treated microbial communities.

Two biocides with similar modes of action were used for this study, namely, GA and an aldehyde-releasing biocide (ARB), bisoxazolidine (3,3′-methylenebis[5-methyloxazolidine]), provided by Schülke & Mayr GmbH. GA is widely applied in oilfield water treatment to control microbial activity, commonly at active concentrations between 25 and 500 ppm; occasionally a 4-h active concentration as high as 2,500 ppm could be required (15–17). Therefore, 100 ppm GA was used in this study to simulate a low-dose scenario, and 750 ppm was used for the high-dose scenario. The same doses of ARB were used according to the recommendation of the supplier, for comparison.

## RESULTS

Anoxic sediment slurries were initially incubated for 16 weeks at 25°C (preenrichment phase). For the experiment, subsamples from the preenrichment were transferred into microcosms containing fresh medium with or without biocides and were incubated for 12 months. The microcosm numbering and biocide treatments are detailed in Table 1. Concentrations of dissolved sulfide, sulfate, and organic carbon amendment (butyrate, propionate, and acetate) were monitored periodically, and samples for nucleic acid assays were frequently collected throughout the experiment. Altogether, we assessed the effectiveness of souring control against the souring microbiota using different biocide treatments, and we investigated how the microbial community responded to the treatments in abundance, diversity, and structure.

**TABLE 1:** Setup details of the experimental microcosms

Microcosm no.	Biocide treatment	Category	Group name
1, 2, 3, 26	No biocide	Test	NB
4, 5	No biocide	Autoclaved	NBa
6, 7, 8	100 ppm GA	Test	GA100
9, 10	100 ppm GA	Autoclaved	GAa100
11, 12, 13	750 ppm GA	Test	GA750
14, 15	750 ppm GA	Autoclaved	GAa750
16, 17, 18	100 ppm ARB	Test	ARB100
19, 20	100 ppm ARB	Autoclaved	ARBa100
21, 22, 23	750 ppm ARB	Test	ARB750
24, 25	750 ppm ARB	Autoclaved	ARBa750

### Effect of biocide treatment on souring control.

**FIG 1:**
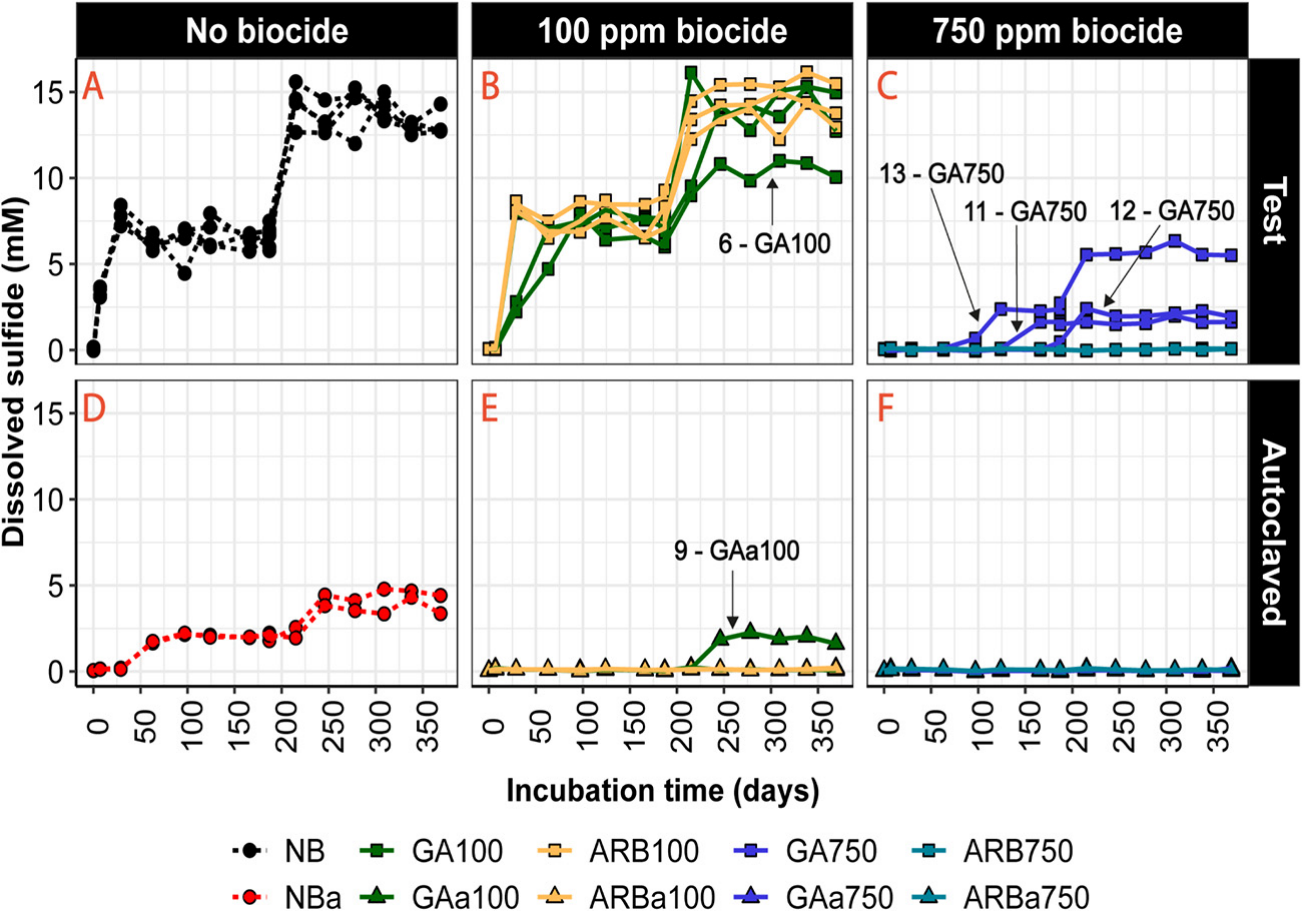
Dissolved sulfide concentrations. (A, B, and C) Test microcosms. (D, E, and F) Respective autoclaved controls. (A and D) NB controls. (B and E) Microcosms treated with 100 ppm biocide. Green lines and green symbols indicate GA100. Yellow lines and yellow symbols indicate ARB100. (C and F) Microcosms treated with 750 ppm biocide. Blue lines and blue symbols indicate GA750. Cyan lines and cyan symbols indicate ARB750. Replicates referred to specifically in the text are pointed out by arrows, and the arrow labels indicate microcosm number followed by group name.

In this study, the effectiveness of souring control was assessed based on the concentration and build-up rate of dissolved sulfide and on the time until souring control began to fail (i.e., dissolved sulfide began to be detected). Souring control was deemed to be effective if no sulfide accumulation could be detected. In the no-biocide (NB) group, the sulfide concentration rose above the detection limit in less than 7 days of incubation. The sulfide concentration reached ∼3.5 mM on day 7 and stabilized at ∼8 mM after day 29 (Fig. 1A). As soon as the additional substrates were introduced into the microcosms on day 187, the sulfide concentration rapidly increased to the second plateau of ∼14 mM, and it remained at that level until the end of the experiment (Fig. 1A). In both of the 100-ppm-biocide-treated test groups (ARB100 and GA100), the sulfide concentration was below the detection limit on day 7. However, by the next sampling time point (29 days of incubation), all microcosms in ARB100 (Fig. 1B) showed over 8 mM sulfide, which was similar to the NB microcosms. From then onward, sulfide concentrations in ARB100 stayed similar to those in NB at all times. The sulfide concentration in GA100 was significantly lower than that in ARB100 and NB on day 29 (*P* < 0.05). However, after 63 days of incubation, the sulfide concentration in GA100 was ∼6 mM, a level comparable to those in NB and ARB100. For the rest of the experiment, sulfide concentrations in GA100 followed a trajectory similar to that of NB (Fig. 1B). Therefore, 100 ppm of either GA or ARB was able to control souring for a short period. However, sulfide accumulated more slowly in GA100 (>0.6 mM/day) than in ARB100 (>1.2 mM/day) after failure.

In ARB750, the sulfide concentration remained below the detection limit throughout the 12-month experiment, indicating lasting effectiveness of souring control (Fig. 1C). In contrast, the sulfide concentration in GA750 remained below the detection limit for the first 2 months for all replicates and then the three replicates showed increasing levels of dissolved sulfide, with levels becoming detectable after day 97 (no. 13), day 166 (no. 11), and day 187 (no. 12); all finally stabilized after day 215 at approximately 3 ± 2 mM (Fig. 1C). Therefore, the treatment with 750 ppm GA achieved only temporary souring control, although the final concentration of sulfide on day 369 was significantly lower in GA750 than in NB (*P* < 0.001).

In the autoclaved no-biocide (NBa) controls, sulfide was detected in all replicates after day 29. The concentration stabilized at ∼2 mM before substrate addition on day 187 and stabilized at the final plateau concentration of ∼4 mM after day 215 (Fig. 1D). In GAa100, one of the replicates (no. 9) showed ∼2 mM sulfide after day 246, which was maintained for the rest of the experiment (Fig. 1E). In all other autoclaved, biocide-treated controls (ARBa100, GAa750, and ARBa750), dissolved sulfide remained below the detection limit throughout the experiment (Fig. 1E and F). These results indicate that autoclaving alone was not sufficient to kill sulfidogenic microorganisms, but the combination of autoclaving and biocide was effective in controlling souring for the 12 months of incubation in all cases except for one replicate of GAa100.

### Effect of biocide treatment on microbial abundance.

**FIG 2:**
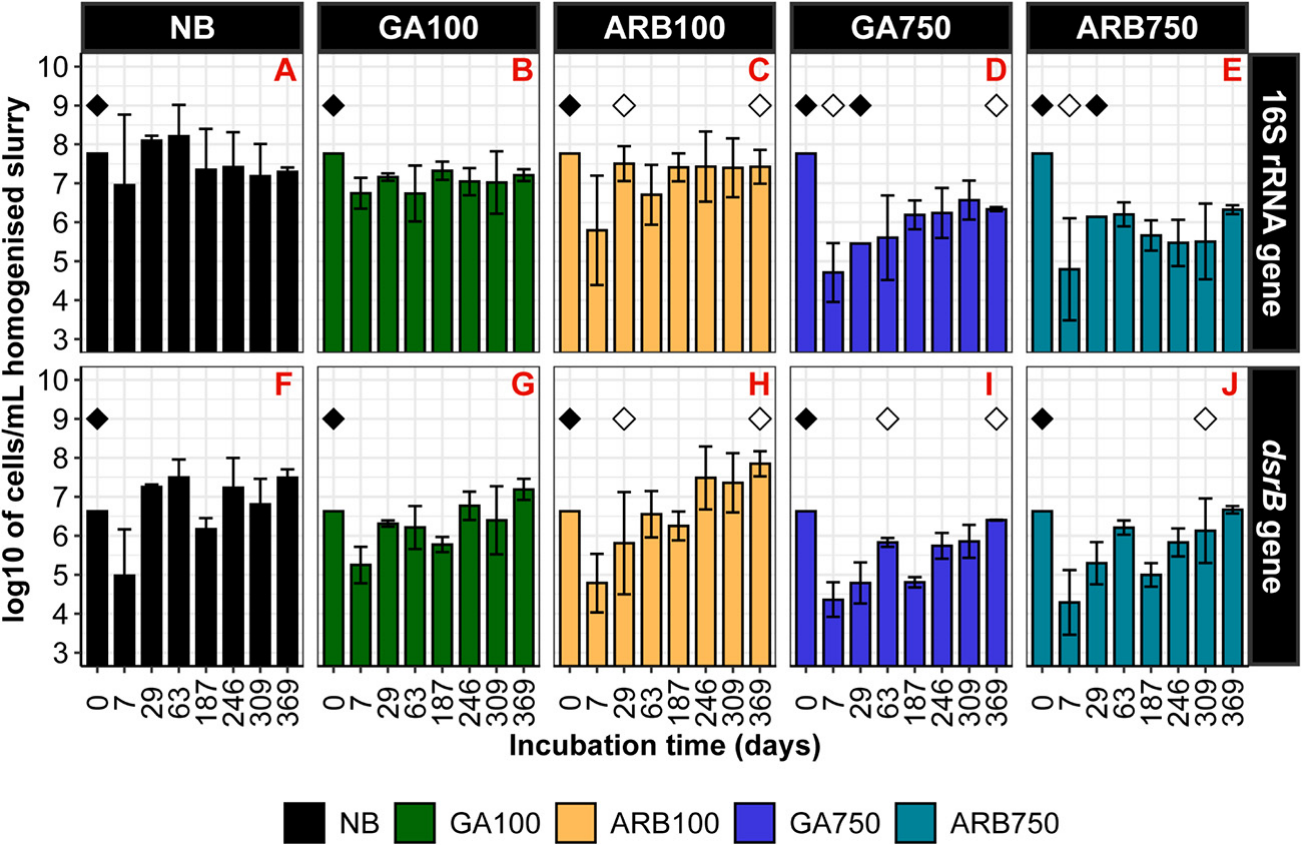
Log_10_-transformed total microbial abundance (A to E) and SRM abundance (F to J) estimated for each test group based on 16S rRNA gene and *dsrB* gene qPCR results, respectively. (A and F) NB. (B and G) GA100. (C and H) ARB100. (D and I) GA750. (E and J) ARB750. Samples with target gene abundances below the detection limit were excluded from the subsequent plotting. Values shown are the averages of microcosm replicates, and the error bars represent the standard deviation among the replicates; *n* = 3 for all bars except for the bars indicated by open diamonds (*n* = 2) or by closed diamonds (*n* = 1). The day 0 values in all panels are the same, referring to the microbial abundance obtained for the inoculum.

To assess the response of the microbial abundance to the biocide treatments, quantitative PCR (qPCR) quantification of 16S rRNA genes and *dsrB* genes was carried out on DNA extracted from the nucleic acid samples collected on days 0, 7, 29, 63, 187, 246, 309, and 369. In NB, the total microbial abundance across all time points was mostly between 10^7^ and 10^9^ cells/ml (Fig. 2A). Similar levels of total microbial abundance were observed in GA100 and ARB100 (Fig. 2B and C), which is congruent with the lack of long-term souring control with these treatments. In contrast, the total microbial abundance for GA750 and ARB750 across all time points was mostly between 10^5^ and 10^7^ cells/ml. The all-time averages of the total microbial abundance for GA750 and ARB750 (log_10_-transformed) were significantly lower than those for NB, ARB100, or GA100 (*P* < 0.01) (Fig. 2). Further, between day 0 and day 7, the total microbial abundance in GA750 and ARB750 was reduced by 3 orders of magnitude, resulting in significantly lower total microbial abundance on day 7 than on day 0 for the two treatments (*P* < 0.05); in NB, GA100, and ARB100, such reductions in total microbial abundance were not clearly observed. Between GA750 and ARB750, no significant log_10_-transformed difference was detected in the all-time average total microbial abundance or in the total microbial abundance at any specific time point, despite their different long-term souring control behaviors (Fig. 1D and Fig. 2D and E). In NBa, the total microbial abundance was mostly between 10^5^ and 10^7^ cells/ml across all time points, similar to values for GA750 and ARB750 (see Fig. S1A in the supplemental material). For autoclaved controls that also received biocide, both 16S rRNA gene levels and *dsrB* gene levels were mostly below the detection limit (equivalent to ∼10^4^ cells/ml) or showed only one replicate above the detection limit (see Fig. S1B to E and G to J). It is worth noting that GAa100 microcosm no. 9 showed indications of biocide failure from day 246 (Fig. 1E), which is consistent with the detection of 10^5^ to 10^7^ cells/ml in this microcosm at the same time points (see Fig. S1B).

The general pattern of SRM abundance in the test and autoclaved groups was similar to that of the total microbial abundance. The SRM abundance in NB was mostly between 10^6^ and 10^8^ cells/ml across all time points; this was about 1 order of magnitude lower than the total microbial abundance (Fig. 2A and F). The all-time average SRM abundance in GA100 and ARB100 was not significantly different from that in NB, while GA750 and ARB750 revealed 1-log_10_ lower all-time average SRM abundance (*P* < 0.01) (Fig. 2F to J). NBa also showed 1-log_10_ lower all-time average SRM abundance, in comparison to NB (Fig. 2F; also see Fig. S1F). As mentioned above, the SRM abundance in the autoclaved and biocide-treated control groups was mostly below the detection limit (see Fig. S1G to J).

### Effect of biocide treatment on microbial community structure.

Sequencing of 16S rRNA genes was performed on the same DNA extracts as used for qPCR assays, thus generating a total of 207 libraries to assess whether treatments with different biocides and doses affected the diversity and structure of the microbial community throughout the 12-month experiment. Microbial community analyses were based on subsamples of 9,161 sequences from the initial libraries. We investigated the alpha diversity, the phylotype-based beta diversity, and the most abundant taxa to describe and to compare the microbial community diversity and structures.

**FIG 3:**
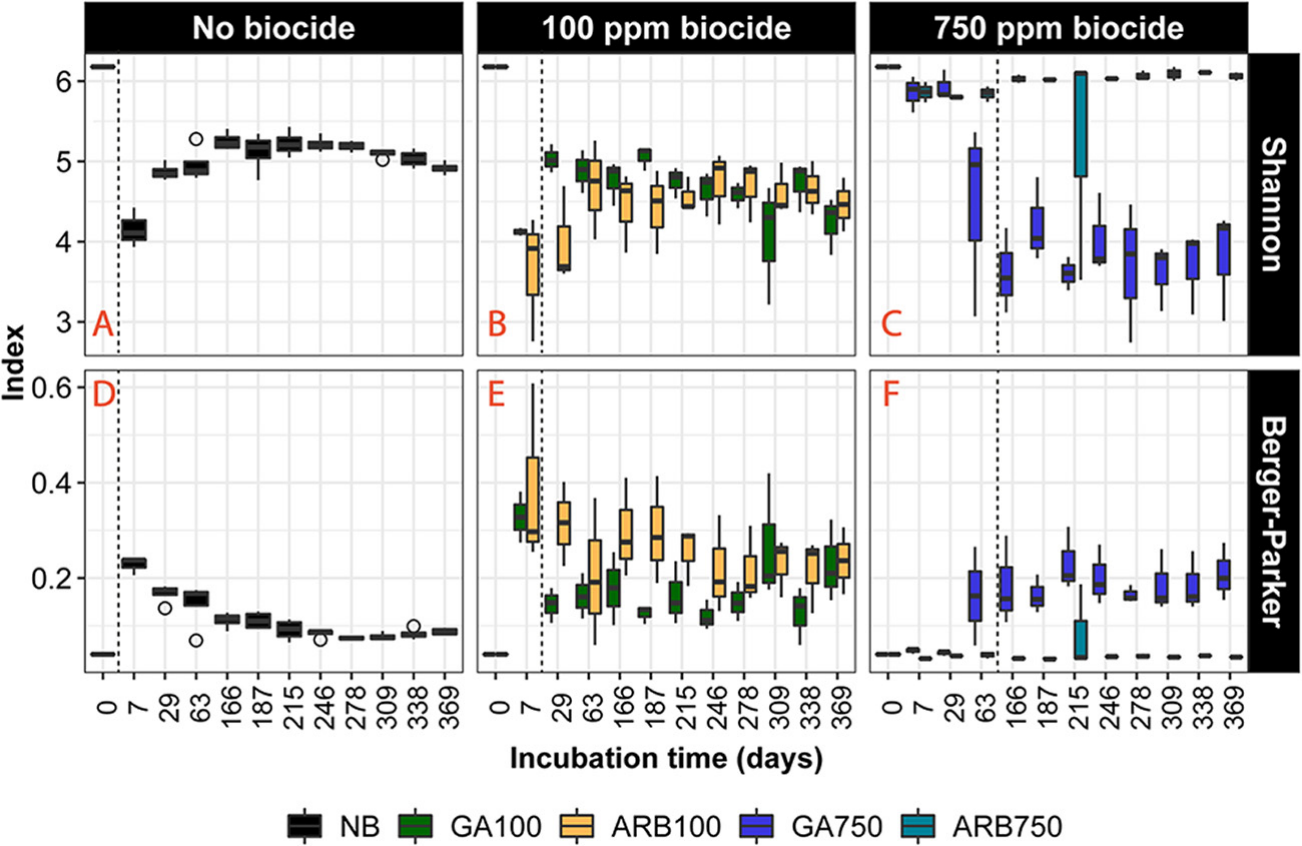
Shannon index (A, B, and C) and Berger-Parker index (D, E, and F) estimated for each test group based on 16S rRNA gene sequencing. (A and D) NB. (B and E) Microcosms treated with 100 ppm biocide. Green boxes indicate GA100, and yellow boxes indicate ARB100. (C and F) Microcosms treated with 750 ppm biocide. Blue boxes indicate GA750, and cyan boxes indicate ARB750. The outliers are indicated by empty circles. The vertical dashed lines in each panel indicate when souring control started to fail in at least one of the replicates of the group(s) in the panel, with the exception of ARB750, which showed no failure of souring control during the experiment.

Alpha diversity of a microbial community, i.e., its richness and evenness, has been associated with its responses to environmental disturbances in a variety of studies (18–20), and such associations are thought to be complex and system specific (21). In this study, the Shannon and Berger-Parker indices were computed to represent alpha diversity, the former to represent the overall alpha diversity of a community, taking into consideration both the richness and the evenness, and the latter to provide insights specifically into the influence of the most dominant microbial taxa on the community evenness (Fig. 3). In NB, the Shannon index first dropped on day 7 and then increased to a plateau, and the Berger-Parker index first increased on day 7 and then dropped to a plateau, both suggesting that the microbial community diversity experienced an initial decrease followed by a process of recovery. However, the final plateau was lower than the initial index on day 0 for the Shannon index and higher for the Berger-Parker index, indicating that the microbial community was less diverse in NB at the later stage of the experiment than at the beginning (Fig. 3A and D). ARB100 and GA100 experienced similar diversity-changing processes, as both the Shannon and Berger-Parker indices for these two groups followed trajectories similar to those of NB. At the later stage, the microbial community was less diverse in ARB100 and GA100 than in NB, as implied by a lower plateau for the Shannon index and a higher plateau for the Berger-Parker index in these two groups (Fig. 3A, B, D, and E).

Until day 63, no pronounced change in the microbial community diversity was observed in either ARB750 or GA750, as both the Shannon and Berger-Parker indices remained similar to the initial values at day 0 (Fig. 3C and F). Thereafter, the two indices continued to remain similar to the initial values in ARB750, while the Shannon index dropped and the Berger-Parker index rose to a new plateau in GA750, congruent with the increase in sulfide concentration (Fig. 1C and Fig. 3C and F). These results indicate that, after day 63, while the microbial community diversity in ARB750 continued to remain stable (and souring remained controlled) (Fig. 1C), the microbial community diversity in GA750 decreased without showing any further recovery, as observed in NB, ARB100, and GA100 (Fig. 3A to F), despite rising souring levels.

The microbial community for autoclaved controls was initially less diverse in NBa than in NB, as implied by a lower Shannon index and a higher Berger-Parker index in NBa than in NB on day 7 (16S rRNA gene sequencing data were not available for NBa on day 0 due to insufficient reads in the library) (Fig. 3A and D; also see Fig. S2A and D). The microbial community diversity recovered in NBa after day 166, as indicated by an increased Shannon index and a decreased Berger-Parker index. However, at the end, the microbial community in NBa was less diverse than those in NB, ARB100, and GA100, as a lower Shannon index and a higher Berger-Parker index were seen in NBa (Fig. 3A, B, D, and E; also see Fig. S2A and F). The Shannon index increased and the Berger-Parker index decreased after day 246 in GAa100 (see Fig. S2B and E), which was congruent with the detection of sulfide in microcosm no. 9 (GAa100) (Fig. 1E), while no pronounced changes in either index were observed in other autoclaved, biocide-treated control groups (ARBa100, GAa750, and ARBa750) (see Fig. S2C to E and H to J).

**FIG 4:**
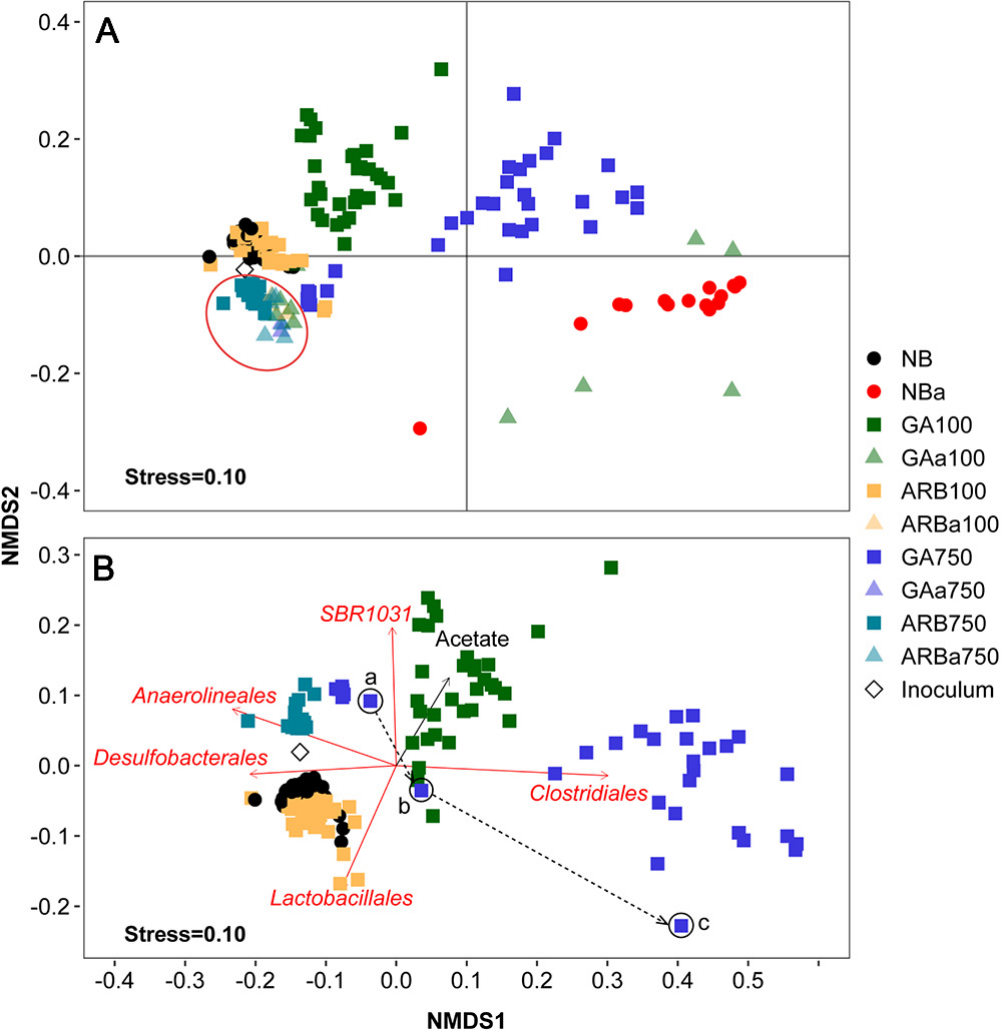
NMDS plot based on weighted UniFrac distances for samples from all groups (A) or from test groups only (B). Each data point represents the position of the corresponding sample on the NMDS plot. In panel A, points representing all samples from ARB750 and the nonsouring samples from autoclaved groups were highlighted with the red oval. In panel B, the points highlighted by the black circles represent microcosm no. 12 (GA750) on day 63 (4 months before souring occurred in this microcosm) (a), microcosm no. 11 (GA750) on day 63 (3.5 months before souring occurred in this microcosm) (b), and microcosm no. 13 (GA750) on day 63 (1 month before souring occurred in this microcosm) (c). The dashed arrow indicates the possible path of the microbial community shift in GA750. The red arrows indicate the correlation between the NMDS coordinate scores and the relative abundances of the most influential taxa (order level), which contributed more than 3% of the microbial community structure differences among the groups. The directions of the red arrows indicate increasing relative abundance. The black arrow indicates the correlation between the NMDS coordinate scores and acetate concentrations. The direction of the black arrow indicates increasing concentrations. The lengths of both the red and black arrows indicate the *R*^2^ of the correlation (a longer arrow indicates a higher *R*^2^).

The phylogenetic analysis-based beta diversity of the microbial community was investigated for all groups. Weighted UniFrac distances were calculated for both the test and autoclaved groups based on the representative sequences for each operational taxonomic unit (OTU) in the libraries after subsampling and were visualized together using a two-dimensional (2D) nonmetric multidimensional scaling (NMDS) plot (Fig. 4A). Most samples had their representative points located in the second and third quadrants of the plot (i.e., all samples from NB, ARB100, GA100, and ARB750 and the nonsouring samples from GA750 and the autoclaved groups). Points representing souring samples from GA750 were located in the first quadrant of the plot, while those representing souring samples from NBa and from GAa100 were located in the fourth quadrant (Fig. 4A). It is worth noting that points representing ARB750 samples partially overlaid those representing nonsouring samples from autoclaved groups, suggesting similar microbial community structures among those samples. This could mean that the microbial community in ARB750 was likely inactive, as also indicated by the lack of sulfide production, volatile fatty acid (VFA) consumption, and changes in alpha diversity (Fig. 1C and Fig. 3C and F; also see Fig. S3C).

To clarify the patterns of microbial community structures of the test groups in detail, a similar investigation into the phylogenetic analysis-based beta diversity of the microbial community was conducted for test groups only, i.e., excluding samples from autoclaved groups from the analysis. Weighted UniFrac distances were calculated for the test groups. Pairwise permutational multivariate analysis of variance (PERMANOVA) confirmed that the samples from each of the five test groups formed unique clusters (*P* < 0.01), which was further visualized using a 2D NMDS plot with a stress value of 0.1 (Fig. 4B). While clusters of NB and ARB100 were both close to the inoculum and partially overlaid each other, GA100 formed a clearly distinct cluster despite behaving similarly to NB and ARB100 in terms of sulfide accumulation, with a short initial delay. This indicates a pronounced difference in the microbial community structure of GA100, compared to those of NB and ARB100. Samples from GA750 formed two subclusters (Fig. 4B). The left subcluster, closer to the experimental inoculum, consisted of only points representing nonsouring samples, which were the samples obtained on day 7 and day 29 (before souring control failed). The points representing souring samples, which were obtained after day 166 (after souring control failed), were clustered to the right of the plot, suggesting a larger community structure dissimilarity, compared to the inoculum. A shift in the community structure from the left subcluster to the right subcluster could be observed for the samples collected on day 63 (Fig. 4B, points a to c), which is discussed in more detail below.

The concentrations of dissolved sulfide, sulfate, butyrate, propionate, and acetate were fitted to the NMDS plot, and the acetate concentration was the only metavariable that showed a significant correlation and hence was visualized (fitness of *P* < 0.01 and *R*^2^ ≥ 0.2 and with the fitted arrow toward the GA100 cluster) (Fig. 4B).

To identify the driving taxa (order level) for the clustering, pairwise similarity percentage (SIMPER) tests were performed on the relative abundance of all taxa among the test groups. The taxa that contributed more than 3% of the microbial composition variance between any test group pair further went through the Kruskal-Wallis test to assess whether the observed variance was significant (*P* < 0.05). The taxa identified as significantly different among the test groups were fitted to the NMDS plot, and only the taxa with fitness *P* values of ≤0.01 and *R*^2^ values of ≥0.35 are presented in Fig. 4B. The fitted arrow for *Clostridiales* pointed to the right cluster formed by the souring samples from GA750 (*R*^2^ = 0.88), suggesting that the clustering of the souring samples in GA750 was strongly driven by *Clostridiale*s (Fig. 4B). The fitted arrow for *Desulfobacterales* pointed in the reverse direction of the arrow for *Clostridiales*, toward the clusters mainly formed by samples that experienced no GA treatment or the nonsouring samples from GA750 (*R*^2^ = 0.42) (Fig. 4B). The ARB100 cluster was mainly influenced by *Lactobacillales* (*R*^2^ = 0.36) and the ARB750 cluster by *Anaerolineales* (*R*^2^ = 0.59) (Fig. 4B). As no known lineage of SRM belongs to these two taxa, this may suggest that the microbial community change associated with ARB treatment was not driven by SRM populations. The GA100 cluster was driven by SBR1031 (*R*^2^ = 0.38) (Fig. 4B).

**FIG 5:**
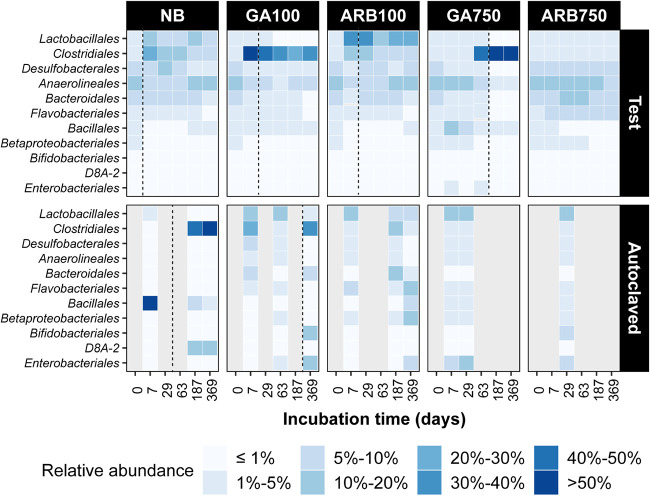
Relative abundance for microbial taxa at the order level in each test and autoclaved group, based on 16S rRNA gene sequencing. The relative abundance for each group is the average relative abundance of all replicates from that group. Only orders showing more than 9% relative abundance at any time point in at least group are included in the figure. Gray columns indicate an absence of data due to insufficient sequencing reads. The vertical dashed lines indicate when souring control started to fail in at least one of the replicates in the corresponding group.

The relative abundance of the most abundant taxa at the order level was investigated to assess whether the taxa contributing the most to the microbial composition variance (Fig. 4) were also the most abundant taxa in a community (Fig. 5). The three taxa with the greatest relative abundance in the experimental inoculum were *Anaerolineales* (10%), *Desulfobacterales* (9%), and *Bacteroidales* (5%). A shift of the most abundant taxa was observed in NB, ARB100, and GA100 on day 7 (Fig. 5), almost congruent with the sulfide accumulation. On day 7, the most abundant taxa in NB became *Clostridiales* (∼24%) and *Lactobacillales* (∼18%). The same two taxa also became the most abundant taxa in ARB100, with *Lactobacillales* achieving a greater relative abundance of ∼40% and *Clostridiales* achieving a lower relative abundance of ∼17%. In comparison, *Clostridiales* (∼53%) stood out as the sole most abundant taxon in GA100 (Fig. 5). On day 187, further shift of the most abundant taxa was observed in NB, with *Anaerolineales* becoming the most abundant taxon (∼17%) while the relative abundance of *Clostridiales* and *Lactobacillales* both dropped to below 10%. An increase in relative abundance for *Anaerolineales* (to ∼13%) and decreases for *Clostridiales* (to ∼7%) and *Lactobacillales* (to ∼20%) were also observed in ARB100, but *Lactobacillales* remained the most abundant taxon. In GA100, the similar increase in relative abundance for *Anaerolineales* was not observed and *Clostridiales* remained the most abundant taxon (∼30%) (Fig. 5).

In ARB750, although the relative abundance of *Bacteroidales* increased to ∼10% on day 29, no major shift in the most abundant taxa was observed throughout the experiment (Fig. 5). In GA750, while the relative abundance of *Anaerolineales* and *Bacteroidales* increased to ∼16% and ∼9%, respectively, on day 7, the major shift in the dominant taxa occurred on day 63, which was congruent with the souring control failure in this group. *Clostridiales* became the most abundant taxon at that time, with a relative abundance of ∼46%, and it remained the most abundant taxon until the end of the experiment, with a further increased relative abundance of ∼70% by day 369 (Fig. 5). For the autoclaved groups showing souring, *Bacillales* represented 83% of the microbial abundance in NBa on day 7; from day 187 onward, the most abundant taxon shifted to *Clostridiales* (50% to 60% relative abundance), while *Bacillales* became less than 5% (Fig. 5). In GAa100, *Clostridiales* also appeared to be the most abundant taxon by the end of the experiment on day 369, but *Bacillales* was not the most abundant taxon at any time (Fig. 5).

**FIG 6:**
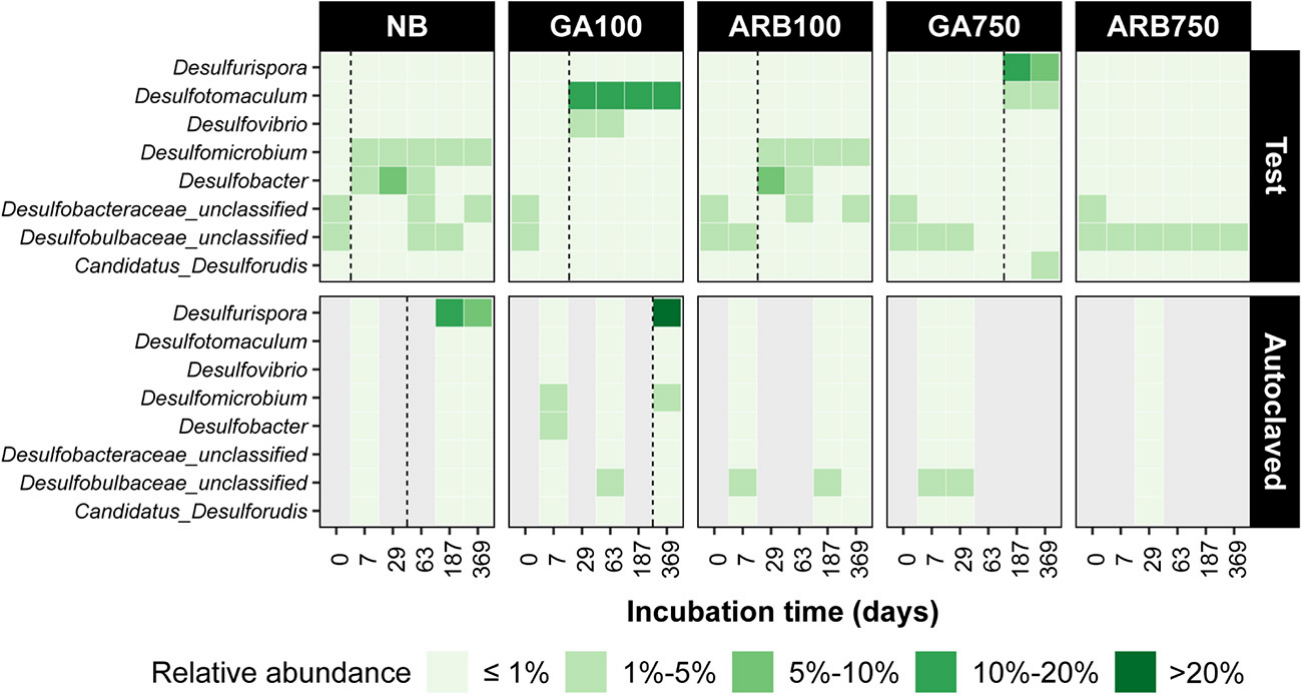
Relative abundance for all SRM at the genus level in each test and autoclaved group, based on *dsrB* gene sequencing. The relative abundance for each group is the average relative abundance of all replicates from that group. Only genera showing more than 2% relative abundance at any time point in at least one group are included in the figure. Gray columns indicate an absence of data due to insufficient sequencing reads. The vertical dashed lines indicate when souring control started to fail in at least one of the replicates in the corresponding group.

To understand the responses of SRM to different treatment conditions, the relative abundance of SRM genera in the total microbial community was investigated (Fig. 6). At the start of the experiment, the most abundant SRM were two unclassified genera affiliated with the families *Desulfobulbaceae* and *Desulfobacteraceae* in the experimental inoculum. However, neither of their relative abundances was above 3%. The sum of relative abundances for SRM at the genus level in the inoculum was less than 9%, and the majority were affiliated with *Desulfobacterales*. In NB, the sum of relative abundances of all SRM varied between 5% and 15% throughout the experiment. *Desulfomicrobium* and *Desulfobacter* became the most abundant SRM from day 7 and remained so until day 63 (Fig. 6). However, their individual relative abundances were always below 7% during this period. Similarly, *Desulfomicrobium* and *Desulfobacter* became the most abundant SRM in ARB100, also with no more than 7% relative abundance for either of them. However, this shift happened by day 29 rather than day 7 (Fig. 6), which was consistent with the same delay in the rise of sulfide concentrations in ARB100 (Fig. 1). In both NB and ARB100, no SRM with individual relative abundance of more than 5% could be identified after day 187. In ARB750, there was no clear shift in the most abundant SRM, compared to the inoculum, throughout the experiment (Fig. 6). This is likely because the microbial community in ARB750 was largely inactive, as suggested by sulfide and VFA profiles.

In GA-treated groups, however, the relative abundance profile for SRM was different from that of NB and ARB-treated groups. In GA100, *Desulfotomaculum* was the main SRM from day 29 and throughout the rest of the experiment, with relative abundance varying between 12% and 17% of the total microbial community. In addition to *Desulfotomaculum*, the relative abundance of *Desulfovibrio* increased shortly to ∼3%, but this was observed only on days 29 and 63 (Fig. 6). In contrast, a major shift of the SRM population was observed on day 187 in GA750, with the relative abundance of *Desulfurispora* rising to 13%. Until the end of the experiment, *Desulfurispora* retained more than 7% relative abundance. After day 187, the relative abundance of *Desulfotomaculum* also increased, as observed in GA100. However, its relative abundance stabilized at ∼3%, which was lower than that observed in GA100. *Desulfurispora* was also detected in the autoclaved groups showing souring, i.e., in NBa (11% on day 187 and 7% on day 369) and GAa100 (30% on day 369) (Fig. 6).

## DISCUSSION

### Souring could be controlled for several months after a single dose of biocide.

In this study, we employed 12-month anaerobic microcosm incubations to evaluate the long-term effects of different biocide treatments on a souring community. We first assessed how long souring could be controlled after a single dose of ARB or GA at 100 or 750 ppm, i.e., how long the sulfide concentration in the aqueous phase was maintained below the detection limit. In NB, souring occurred within 7 days after substrates were added on day 0. In comparison, souring control was achieved to a certain extent in all four biocide treatment scenarios, as the sulfide concentration stayed below the detection limit for longer than in NB. In the two low-dose treatment scenarios (ARB100 and GA100), souring control was only temporary and failed after day 7, as indicated by the increase in the sulfide concentration (Fig. 1B), which followed a similar trajectory and reached a comparable final plateau concentration, compared to NB (Fig. 1A). GA100 showed better control than ARB100, as the increase in the sulfide concentration after day 7 was slower for GA100 than for ARB100. However, microbial community changes in GA100 were significant and might have implications for longer-term biocide treatment strategies, as discussed below.

A possible explanation for the early failure of both biocides at 100 ppm is that the concentration of the biocide was not maintained in the microcosms and dropped below an effective dose. For example, there have been multiple reports of rapid GA removal due to sediment adsorption, formation of hydrates, biotic degradation, or covalent binding to microbial cells in various cases in which up to 250 ppm GA was used to treat water or sediments consisting of environmental microbiota (13, 22, 23). GA concentrations were not assessed in the present study; however, ARB concentrations were. In ARB100, the residual concentration of the biocide dropped to 13 ppm after 7 days, a reduction of more than 80%. On day 29, the concentration was below the detection limit of 11 ppm (Table 2). In contrast, the residual concentrations of ARB in the corresponding autoclaved control (ARBa100) were 50 ppm and 38 ppm on days 7 and 29, respectively (Table 2), suggesting that the decrease in the ARB concentration in ARB100 could be due to both biotic and abiotic processes and the rapid depletion of ARB was likely associated with the souring control failure in ARB100. It is known that ARB is a sulfide scavenger; therefore, in addition to the processes mentioned above for GA, another possibility is that a low dose of ARB may be insufficient to inhibit the growth of SRM before sulfide accumulates to a level that eliminates the biocide from the medium.

**TABLE 2:** Residual ARB concentration and ARB concentration reduction measured after 7 days and 29 days for each ARB-treated group

Parameter and group^*a*^	Day 7	Day 29
Residual ARB concn (ppm)		
ARB100	13	<11
ARBa100	50^*b*^	38^*c*^
ARB750	444	312
ARBa750	503^*b*^	412^*b*^
ARB concn reduction (%)		
ARB100	87	>89
ARBa100	50	62
ARB750	41	58
ARBa750	33	45

a The residual concentration presented is the mean concentration among triplicates unless otherwise specified. The detection limit was 11 ppm for all measurements. The concentration reduction presented is the percentage of the concentration reduction relative to 100 ppm (ARB100 and ARBa100) or 750 ppm (ARB750 and ARBa750).

b Based on the average measurements for the available duplicates.

c Based on the measurement for the only available sample.

In the two high-dose treatment scenarios (ARB750 and GA750), souring control was achieved for considerably longer periods. In ARB750, no sulfide was observed throughout the 1-year experiment (Fig. 1C). For GA750, souring was controlled for approximately 3, 5.5, and 6 months in microcosms 13, 11, and 12, respectively, and resulted in lower final sulfide concentrations (<5 mM) than those in NB, ARB100, and GA100 (>10 mM) (Fig. 1A and B). The extended duration of souring control for both high-dose treatments indicates that an initial loss of biocide did not immediately compromise the treatment, i.e., the biocide concentration was not immediately reduced below an effective dose. In ARB750, the residual biocide concentrations on days 7 and 29 were 444 and 312 ppm, respectively, thus reaching 58% reduction by day 29 (Table 2). This is again a greater loss than in the respective autoclaved control, ARBa750, where the loss was up to 45% during the same period. Even if further loss occurred at later times, the amount available was sufficient to inhibit SRM activity and general microbial growth in both ARB750 and ARBa750. In GA750, the delayed occurrence of souring and reduced severity suggested that the initial GA dose of 750 ppm might eventually have been reduced below an effective dose or that the *de facto* concentration was able only to alleviate souring, rather than to control it. However, this could still be a practically acceptable long-term souring control outcome.

It is worth mentioning that souring was observed in some of the autoclaved microcosms, i.e., all NBa microcosms and one of the GAa100 microcosms (no. 9) (Fig. 1D and E). The occurrence of souring in these autoclaved microcosms was consistent with the increase in the relative abundance of *Desulfurispora* (Fig. 6), a sulfate-reducing genus known to form endospores (24). Depletion of propionate and butyrate was also observed in the same autoclaved microcosms (see Fig. S3D and E in the supplemental material). Therefore, it is likely that endospore-forming SRM, such as *Desulfurispora*, survived the autoclaving process and gradually recovered to resume sufidogenesis over time. Previous studies based on estuarine sediment enrichments from southwest and northeast England, including from the River Tyne, showed that these sediments host thermophilic bacterial spores that are able to survive high-temperature treatment, including multiple autoclaving cycles, and resume sulfate reduction activity (25, 26). Our experiment shows that autoclave-resistant mesophilic SRM are also present in River Tyne sediment and that the combination of autoclaving and GA or ARB treatment was effective in inhibiting their growth for the duration of the experiment, with the exception of one replicate of GAa100.

In addition to sulfide measurements, the concentrations of VFAs (i.e., butyrate, propionate, and acetate) and the sum of sulfur concentrations (i.e., the sulfate concentration plus the sulfide concentration) were monitored to more thoroughly assess the microbial activity supporting souring (see Fig. S3). Minimal VFA consumption was observed in most of the autoclaved, biocide-treated microcosms and in ARB750, where no souring occurred throughout the experiment (see Fig. S3C, E, and F), whereas VFAs were generally depleted in the souring samples from ARB100, GA100, GA750, NB, and NBa and one replicate of GAa100 (no. 9) (see Fig. S3A to D). Hence, souring control was likely achieved by at least partially suppressing the sulfate reduction pathways associated with butyrate, propionate, or acetate consumption (see Fig. S3). Further, a greater final sum of sulfur concentrations was observed in the groups that experienced no souring (i.e., ARB750, ARBa100, GAa100, ARBa750, and GAa750) than in those that experienced souring (see Fig. S4), suggesting less loss of sulfur into chemical species other than aqueous sulfide or sulfate in the nonsouring groups. This could be explained by lower assimilation rates in nonsouring groups due to expectedly lower microbial activity. Meanwhile, as the sum of sulfur concentrations referred to in this study did not consider all possible sulfate reduction intermediate products, such as sulfite and thiosulfate (27), the greater final sums of sulfur concentrations in nonsouring groups could also imply that the effectiveness of the biocide was achieved by interfering with the complete process of sulfate reduction in the microbial community, rather than by introducing incomplete sulfate reduction through merely blocking the final stage of sulfidogenesis while allowing sulfate reduction into intermediate products.

### Both the dose and the type of biocides showed effects on the abundance, diversity, and structure of the microbial community.

While the influence of the biocide type on the microbial abundance was less obvious, the influence of the biocide dose was clear. The short-term influence of biocide dose could be observed as the high-dose groups (ARB750 and GA750) experienced an initial disruption of microbial growth, implied by the significant log_10_ reductions in the total microbial abundance and SRM abundance between day 0 and day 7, as opposed to the less pronounced initial abundance reduction in the low-dose groups (ARB100 and GA100). A comparable reduction in microbial abundance was also observed for the first 7 days in a similar batch study in which 100 ppm GA was dosed against a stream microbial community (13). The initial disruption of microbial growth by a biocide observed across different studies suggests that the first days after the introduction of a biocide may be considered the critical response phase for the biocidal action against the microbial growth, probably because the *de facto* biocide concentration is expected to be at its peak during this phase. Our study also shows that, besides short-term effects, biocide dose also had a long-term effect, as the overall microbial abundance for ARB750 and GA750 remained lower than that for ARB100 and GA100 throughout the experiment (Fig. 2). It is important to note that such a seemingly dose-dependent suppression effect on microbial abundance does not indicate that the higher dose necessarily leads to a lower abundance of viable cells. A recent study using cultivable plate counts for quantification suggested that, while the biodegradation activity of the community recovered after exposure to GA, no difference in microbial abundance of viable cells could be identified before and after GA exposure at up to 300 ppm for 26 days (28). Since GA in solution is expected to be consumed as it cross-links with the cell wall regardless of the viability status of the bacteria (29), it would be valuable to further explore the dynamics between the abundance of viable and nonviable cells when facing a biocide and to clarify whether nonviable cells of a microbial community may “protect” the viable cells by consuming the biocide, thus undermining the performance of the biocide treatment.

The type and dose of the biocide together played critical roles in the overall microbial community structure, as distinct clusters driven by different taxa were formed for different treatment scenarios, as seen in the NMDS plot (Fig. 4). The clusters for NB and ARB100 were close to each other, and *Lactobacillales* was the main driver to separate these two clusters, indicating that ARB100 favored *Lactobacillales* over other microbial populations. This was further supported as *Lactobacillales* was the most abundant taxon throughout the experiment in ARB100, while in NB it was less abundant than *Clostridiales* during the early stage (days 7, 29, and 63) and than *Anaerolineales* during the late stage (days 187 and 369) (Fig. 5). Species of *Lactobacillales* have been found to outcompete species of *Clostridiales* for carbon sources when cultured with low concentrations of glucose (30). This study further suggests that *Lactobacillales* may also outcompete *Clostridiales* for VFAs in the presence of low concentrations of ARB. Furthermore, *Anaerolineales* is frequently recovered from the deep biosphere, and a recent comparative genomic study suggested that members affiliated with *Anaerolineales* possess the metabolic potential of both fixing CO_2_ through the Wood-Ljungdahl pathway and using a wide range of exogenous carbon sources such as formate (31). This may help to explain why *Anaerolineales* became relatively more abundant toward days 187 and 369 in NB and ARB100, when the added VFAs were depleted and CO_2_ or formate presumably became increasingly available. Finally, taxa known to be fermentative rather than capable of sulfate reduction, such as *Lactobacillales* and *Anaerolineales*, also showed clear influences on the microbial community structure in response to different biocide treatments, as they both drove some of the clusters in the NMDS plot (Fig. 4B) and affected the relative abundance profiles in different groups (Fig. 5). Thus, the fermenter populations likely played a role in the efficacy of the souring control, even though they were not directly associated with sulfide production.

For ARB750, the NMDS cluster appeared to be mainly driven by *Anaerolineales* (Fig. 4). However, ARB750 revealed a static relative abundance profile, with no pronounced relative abundance changes being observed from any taxon throughout the experiment, compared to the experimental inoculum. Thus, the lasting effect of high-dose ARB more likely favored no specific taxon, and the observation that *Anaerolineales* was the driving taxon for the ARB750 cluster in the NMDS plot could be attributed to the decrease in relative abundance of *Anaerolineales* in other groups. Considering that ARB750 also showed a lack of sulfide accumulation or substrate consumption (Fig. 1; also see Fig. S3), a lack of change in diversity (Fig. 3), and a microbial community structure similar to those of the autoclaved and biocide-treated groups (Fig. 4), we conclude that microbial activity in ARB750 was largely restricted.

The GA100 cluster was driven by SBR1031 (Fig. 4), an uncultivated lineage whose members were found to contain key genes for acetogenic dehydrogenation according to a metagenomics study (33). This could be associated with the observed acetate accumulation in microcosm no. 6 (GA100) due to the expected suppression of acetate-dependent sulfate reduction pathways, as discussed above (see Fig. S3B). GA750 formed two distinct subclusters, indicating a pronounced shift of the microbial community structure during the experiment (Fig. 4B). GA treatment seemed to favor *Clostridiales* in the long term regardless of the dose, as *Clostridiales* became the most abundant taxon by the end of the experiment in both GA100 and GA750. Furthermore, the surge in relative abundance of *Clostridiales* was observed ∼1 month before souring control failed in both GA100 and GA750, indicating a connection between high relative abundance of *Clostridiales* and the upcoming souring with GA treatment (Fig. 5).

In this study, the two main SRM-containing orders identified (*Desulfobacterales* and *Clostridiales*) appeared to drive the clusters to opposite directions in the NMDS plot, with the ARB clusters being more influenced by *Desulfobacterales* and the GA clusters being more influenced by *Clostridiale*s (Fig. 4). This suggests that ARB and GA favored different SRM taxa in the long term. ARB100 showed a SRM relative abundance profile similar to that of NB over time (Fig. 6). In these two groups, the main SRM genera showing increases in relative abundance during the experiment were *Desulfobacter* and *Desulfomicrobium* (Fig. 6), both affiliated with *Desulfobacterales*. *Desulfobacter* is capable of utilizing acetate (34); *Desulfomicrobium* is capable of utilizing a wide range of fermentation products, such as formate and H_2_/CO_2_, which could be derived from the fermenters identified in these groups (35). Other SRM present at lower relative abundance were typically propionate and butyrate consumers, which were not reported to utilize acetate or other low-molecular-weight fermentation products. ARB750 revealed a SRM relative abundance profile similar to those identified in the nonsouring autoclaved control samples (ARBa100, GAa750, and ARBa750), probably due to the highly restricted microbial activity in this group, as discussed above.

For GA, the SRM community structure became distinguishable from that of NB in both the high- and low-dose scenarios. In GA100, the sulfate-reducing populations experiencing increases in relative abundance were mainly *Desulfotomaculum* and, to a lesser extent, *Desulfovibrio* (Fig. 6), which were not the same as the favored SRM identified in ARB100 (*Desulfobacter* and *Desulfomicrobium*). Thus, the susceptibility of SRM to the biocides was likely dependent on the type of biocide being used. Furthermore, while ARB resistance remains poorly understood, GA resistance of bacteria has been found to be mediated through increases in efflux pumps, which enhance the rate of biocide export (36), and it has been postulated that the mechanism to control osmotic stress retained by halotolerant taxa might be a key genetic trait promoting GA resistance (13). This may help to explain the relative abundance increases for *Desulfotomaculum* and *Desulfovibrio* in GA100, as halotolerant species are not uncommon for these two SRM genera (37–40).

Another mechanism for microbes to resist environmental stresses such as biocides is to enter dormancy and to form spores (21, 41), and a study suggested that the spore coat is able to protect spores against killing by GA (42). Thus, in GA100, the greater relative abundance of *Desulfotomaculum*, compared to *Desulfovibrio*, might be associated with the different spore-forming abilities of these two SRM genera, because spore formation is absent for *Desulfovibrio* but commonly observed for *Desulfotomaculum* (25, 35, 37, 43). In GA750, while *Desulfotomaculum* was still detected with high relative abundance on days 187 and 369, the most abundant SRM on these days was *Desulfurispora*, another endospore-forming lineage affiliated with *Clostridiales* (24, 44). *Desulfurispora* was also the main SRM lineage in the autoclaved groups in which at least one replicate showed souring (NBa and GAa100 no. 9) (Fig. 6). Thus, the increasing relative abundance of *Desulfurispora* and *Desulfotomaculum* with a high dose of GA might also be linked to the spore-forming ability of these two genera, and *Desulfurispora* might be more resilient than *Desulfotomaculum* in withstanding environmental stresses such as heat or high concentrations of biocide. Further studies are needed to elucidate the reasons for the greater resilience of *Desulfurispora*.

### Application of microbial community analyses to guide field planning of souring control.

As seen in this study, evaluating biocide efficacy against a complex souring microbiota over a long period was able to reveal important insights about the long-term interactions between the biocide treatments and the microbial community. Souring control failed in ARB100, GA100, and GA750 to different extents, with the 750-ppm dose of GA preventing souring substantially longer than the 100-ppm treatments. The long-term succession profiles of the microbial community structure were significantly different among these groups, and distinct SRM taxa became the dominant SRM populations by the end of the 1-year experiment. In practice, this means that different biocide program designs might lead to distinct microbial community structures in the long run. Therefore, when a biocide-based souring control program fails, a predetermined universal remedial plan may not be the best contingency option; instead, the remedial plan could be tailored to the specific microbial community structure that developed as a consequence of the failed treatment program, to achieve more effective remedial performance. Furthermore, greater relative abundance of Gram-positive and spore-forming SRM (i.e., *Desulfotomaculum* or *Desulfurispora*) was observed on day 369 in both GA100 and GA750, suggesting that GA treatment may lead to the enrichment of expectedly more resistant SRM populations even at a low dose, making any further biocide treatments more costly and more damaging for health, safety, and the environment, because higher doses, longer biocide exposure, and more toxic biocides may be required. It is worth highlighting that only ARB750 did not lead to the enrichment of any expectedly resistant SRM populations, while achieving effective souring control over the duration of the experiment. This was likely because the microbial activity was largely restricted throughout the experiment. Thus, selecting the right type and dose of biocide is critical not only for the immediate effect of alleviating sulfide but also for the long-term effect of avoiding an undesirable microbial community succession.

Natural microbial communities have been considered general quantitative sensors for the changes in their surrounding environment because they are sensitive to environmental disturbances (45). In a recent study, we showed that the changes in the abundance and diversity of the microbial community could be indicative of the occurrence of souring during the recovery period after short-term low-dose biocide treatment in sand pack bioreactors (46). In the present study, clear connections between souring control failure and changes in microbial community diversity and structure were also widely observed, over several months and for different biocide types and doses. In ARB100, GA100, and GA750, souring control started to fail following the observation of the initial microbial diversity loss (days 7, 7, and 63, respectively) (Fig. 3). The occurrence of souring control failure also followed the emergence of the long-term dominant taxa (*Lactobacillales* in ARB on day 7, *Clostridiales* in GA100 on day 7, and *Clostridiales* in GA750 on day 63) (Fig. 5). Particularly for GA750, the three points connecting the left subcluster to the right subcluster on the NMDS plot represented samples collected from the three GA750 microcosms on day 63, and souring control failed in these microcosms on days 187, 166, and 97 (Fig. 4B). Therefore, the three points from left to right represented samples collected 4 months, 3.5 months, and 1 month prior to the start of souring control failure in their corresponding microcosms. In other words, before sulfide accumulation became observable, the microbial community structure had already been gradually shifting for months from a subcluster consisting of only nonsouring communities to a subcluster consisting of only souring communities. This shifting process was clearly captured by 16S rRNA gene sequencing and NMDS analyses of the microbial community structure. Overall, this study suggests that the changes in microbial ecological metrics may be used as early warnings of changing souring conditions in oil reservoirs. Frequently monitoring the changes in the microbial ecological metrics of the reservoir microbial community may help to forecast souring control failure, thus allowing necessary remedial strategies to be planned in advance.

## MATERIALS AND METHODS

### Samples, preenrichment, and experimental setup.

Surface sediment from the River Tyne estuary (United Kingdom) (54°57′51″N, 1°40′60″W) was sampled at low tide with a clean trowel into a sterile container, which was immediately sealed to minimize gas exchange. The sediment was kept at 4°C until used for preenrichment to establish a souring community. The souring microbial community enriched from the River Tyne estuary sediment has been frequently used as a model community for investigations into the biodegradation and microbial processes in oil reservoirs (26, 47, 48). The preenrichment consisted of a sediment slurry made with anoxic synthetic brackish water medium (49) in a 3:1 ratio of medium to sediment, prepared as a single batch in a 2-liter glass bottle and subsequently sealed with a butyl rubber stopper under N_2_ flow. The medium was amended with extra carbon substrates (3 mM acetate, 1 mM propionate, and 1 mM butyrate) and contained 7.5 ml of 0.2 M ascorbic solution instead of 7.5 ml of 0.2 M Na_2_S as the reducing agent. Acetate, propionate, and butyrate are widely accepted as major electron donors used by SRM in oil reservoirs (3, 27, 50). They can be produced *in situ* through hydrocarbon biodegradation (51, 52) and have been found to be available in the produced water from locations around the globe (53–57). Acetate, propionate, and butyrate have frequently been used in similar studies investigating oil reservoir souring (50, 58–60) and thus were also selected for this study. The preenrichment was incubated for 16 weeks at 25°C. Souring was considered successfully established after more than 20 mM sulfate was consumed (see Fig. S5 in the supplemental material). The preenrichment was then used as the inoculum for the experiment. To set up the test microcosms and their corresponding autoclaved controls for the experiment, the serum bottles received the autoclavable fraction of the culture medium and then they were capped and sealed. Subsequently, the microcosms were autoclaved for 20 min at 121°C, and the headspaces were exchanged with N_2_:CO_2_ (90:10). The remaining medium constituents and biocides were dispensed aseptically and anaerobically using syringes when the microcosms had cooled to room temperature. The medium used for the experiment was the same anoxic sterile synthetic brackish water medium as used for the preenrichment, amended with organic carbon substrates (3 mM acetate, 1 mM propionate, and 1 mM butyrate) and either (i) no biocide, (ii) GA at a final concentration of 100 ppm, (iii) GA at a final concentration of 750 ppm, (iv) ARB at a final concentration of 100 ppm, or (v) ARB at a final concentration of 750 ppm. For the test microcosms, 30 ml of the souring preenrichment was inoculated into each of the microcosms containing 180 ml of the amended medium. For the autoclaved controls, the souring preenrichment was subsampled, and the subsample was autoclaved for 20 min at 121°C in one batch and cooled to room temperature. Then, 30 ml of the autoclaved subsample of souring preenrichment was inoculated into each of microcosms also containing 180 ml of the same amended medium as their corresponding test microcosms. Each biocide treatment was set up in triplicate, while the NB controls were set up in quadruplicate. The respective autoclaved controls were prepared in duplicate. The complete experimental setup is detailed in Table 1. All 26 microcosms were then incubated at 25°C for 369 days. After 187 days of incubation, 10 mM sulfate, 5 mM acetate, 1 mM propionate, and 1 mM butyrate were added to all 26 microcosms to compensate for the consumption of the substrates.

### Chemical sampling, measurements, and analyses.

To measure the concentrations of dissolved sulfide, sulfate, butyrate, propionate, and acetate, samples were taken from the batch enrichment bottle on days 0, 1, and 2 and then once per week for the next 15 weeks during the preenrichment; during the experiment, samples were taken from each of the microcosms on days 0, 7, and 29 and then approximately every month for a total of 12 months of incubation. For the sampling, the contents were first homogenized by inverting and lightly shaking the enrichment bottle or the microcosms. Sediment slurry (3 ml) was removed using an N_2_-flushed sterile syringe with a 23-gauge sterile needle and was immediately filtered through a 0.2-µm filter. Filtrate (0.1 ml) was immediately added to 1.9 ml of the copper reagent, which consisted of 50 mM HCl and 5 mM CuSO_4_. The aqueous sulfide was then detected as a colloidal solution of copper sulfide using a spectrophotometer (61), and the concentration was determined based on a standard curve. The detection limit was 0.1 mM.

The remaining filtrate was stored at –20°C until further analysis to quantify the concentrations of sulfate, acetate, propionate and butyrate. Anion concentrations were measured using an Integrion ion chromatography system equipped with an AS-AP autosampler (both from Thermo Fisher Scientific, UK). The injection loop was 25 μl, and 10 μl of prediluted sample (1:200 in 18.2-MOhm · cm^−2^ water) was injected onto the analytical column (IonPac AS11-HC, 4 μm; Thermo Fisher Scientific) with the flow rate set to 0.015 ml/min in an oven compartment set to 30°C. The eluent (KOH) was generated automatically via a Dionex eluent generation cartridge and was set to a gradual increase (1 mM from 0 to 5 min, 1 mM to 15 mM from 5 to 14 min, 15 mM to 30 mM from 14 to 23 min, and 30 mM to 60 mM from 23 to 50 min). The peak areas were integrated using Chromeleon v3.7 software.

Selected samples from the ARB-treated test and autoclaved microcosms were measured for residual ARB concentrations, which was performed at Schülke & Mayr GmbH (Hamburg, Germany). The samples first went through steam distillation to release all formaldehyde (FA) molecules bound to ARB. Subsequently, acetylacetone was added in the presence of ammonium acetate to convert FA to 3,5-diacetyl-1,4-dihydrolutidine, and the absorption at 412 nm was measured with a UV/visible spectrophotometer. The concentrations of FA were determined using a FA calibration curve and were divided by 0.47 to convert the values to ARB concentrations (62). All chemical data, i.e., the concentrations of dissolved sulfide, sulfate, acetate, propionate, and butyrate and the residual concentration of ARB, were compared using analysis of variance (ANOVA), followed by Tukey’s honestly significant difference (HSD) *post hoc* tests for pairwise comparison.

### Nucleic acid material sampling and DNA extraction.

For DNA extraction from the souring preenrichment and microcosms, contents were first homogenized by inversion and light shaking. A 10-ml sample of sediment slurry was taken using a sterile syringe with a 23-gauge sterile needle and transferred into a 15-ml sterile centrifuge tube. The samples were then stored at −80°C until further processing. At the end of the preenrichment, one sample was taken from the souring sediment and one from the autoclaved souring sediment as the day 0 benchmarks for the experiment. Throughout the experiment, samples were taken from each of the 26 microcosms on days 7, 29, 63, 166, 187, 214, 246, 277, 309, 337, and 369. Thawed samples were centrifuged at 4,696 × *g* (Heraeus Multifuge X1R; Thermo Fisher Scientific) to pellet the cells. The supernatant was removed, while the wet sediment from each sample was collected with a sterile scoop. DNA was extracted using a cetyltrimethylammonium bromide (CTAB)-based protocol, as described elsewhere (63). The DNA extracts were subsequently used for qPCR assays and 16S rRNA gene sequencing.

### qPCR assays for quantification of total microbial abundance and SRM abundance.

Total microbial abundance was estimated using qPCR assays targeting 16S rRNA genes with primers Ba519F and Ba907R (64). The assays were carried out on a QuantStudio 3 real-time PCR system (Thermo Fisher Scientific), in 10-µl reaction mixtures consisting of 5 µl of JumpStart *Taq* ReadyMix (Sigma-Aldrich, Germany), 3 µl of template DNA, 4.5 mM MgCl_2_, and 500 nM each primer. Thermocycling conditions for the 16S rRNA gene qPCR were as follows: initial denaturing at 96°C for 2 min, followed by 40 quantification cycles of denaturing at 96°C for 30 s, annealing at 52°C for 30 s, and extension at 72°C for 1 min. The abundance of SRM was estimated on the same qPCR system targeting genes encoding the β-subunit of dissimilatory sulfite reductase (*dsrB* genes) with primers DSR1728f-mix and rDSR4r-mix (65). The total reaction mixture was 10 µl, which consisted of 5 µl of SsoAdvanced universal SYBR green supermix (Bio-Rad, UK), 1 µl of template DNA, and 1 µM each primer mix. Thermocycling conditions for the *dsrB* gene qPCR were as follows: initial denaturing at 95°C for 5 min, followed by 40 quantification cycles consisting of denaturing at 95°C for 15 s, annealing at 55°C for 30 s, and extension at 72°C for 20 s. The details of the primer sets are described in Table 3. The specificity of both 16S rRNA gene amplification and *dsrB* gene amplification was verified using melting curves constructed from 60°C to 95°C in 0.2°C steps, with a holding time of 5 s per step. All DNA samples, standards, and no-template controls (NTCs) were assayed in triplicate. To minimize errors introduced by the discrepancies in PCR amplification efficiency between the standards and samples, the one-point calibration (OPC) method was adopted for quantification (66, 67). The raw fluorescence data without baseline correction for each query template were imported into LinRegPCR v2018.0. Via the linear regression of raw fluorescence data (log transformed) against the amplification cycles, baseline subtraction was optimized. The threshold cycle (*C_T_*) and the amplification efficiency (*E*) for each PCR were determined using the software (68, 69). *N*_0,standard_ denotes the known number of target genes in the standards used as the OPC references. In this study, four 10-fold serial dilutions of standards (equivalent to 10^4^ to 10^7^ genes) were used as the OPC references for both 16S rRNA gene and *dsrB* gene OPC calculations. *N*_0,template_ denotes the unknown number of target genes in the query template, which was calculated based on *N*_0,standard_ using equation 1 (67, 69).

(1) $${\text{N}}_{0\text{template}}={\text{N}}_{0\text{standard}}\times \frac{E_{\mathrm{standard}}{}^{C_{T}\mathrm{standard}}}{E_{\mathrm{template}}{}^{C_{T}\mathrm{template}}}$$

The average amplification efficiency values were *E_standard_* = 1.68 ± 0.02 and *E_template_* = 1.70 ± 0.05 for 16S rRNA gene qPCR and *E_standard_* = 1.66 ± 0.01 and *E_template_* = 1.71 ± 0.04 for *dsrB* gene qPCR. The detection limit was ∼10^3^ genes/μl for all 16S rRNA gene and *dsrB* qPCR assays. For each query template, 12 *N*_0,template_ values were produced based on the four OPC reference standards (in triplicate). The final quantification of target genes in any sample was determined as the arithmetic means of all *N*_0,template_ values obtained from the replicate query templates corresponding to that sample. The results were converted to cells per milliliter of homogenized slurry to allow direct comparison between samples. The copy number of 16S rRNA genes per cell was estimated to be 4.9 based on the rrnDB search results, last accessed in May 2019 (70); the copy number of *dsrB* genes per cell was assumed to be 1 (71).

**TABLE 3:** Details of the primers used for qPCR assays

Target gene and primer	Annealing temp (°C)^*a*^	Primer sequence (5′ to 3′)	Size (bp)	Reference^*b*^
16S rRNA	52		407	64
Ba519F		CAGCMGCCGCGGTAANWC		
Ba907R		CCGTCAATTCMTTTRAGTT		
*dsrB*	55		∼355	65
DSR1728f-mix				
DSR1728f-a		CAYACCCAGGGNTGG		
DSR1728f-b		CAYACBCAAGGNTGG		
DSR1728f-c		CATACDCAGGGHTGG		
DSR1728f-d		CACACDCAGGGNTGG		
DSR1728f-e		CATACHCAGGGNTAY		
rDSR4r-mix				
rDSR4r-a		GTGTAACAGTTWCCRCA		
rDSR4r-b		GTGTAGCAGTTDCCRCA		
rDSR4r-c		GTATAGCARTTGCCGCA		
rDSR4r-d		GTGAAGCAGTTGCCGCA		

a The primer annealing temperature was optimized for this work.

b The coverage of the 16S rRNA gene was estimated using TestPrime (http://www.arb-silva.de/search/testprime) (77); allowing one mismatch, the primer sets in this study cover 85.3% of archaea and 93.6% of bacteria (SILVA SSU database release 138, last accessed in March 2020). The coverage of the *dsrB* gene was based on a previous study (65). Allowing one mismatch, the primer sets in this study cover 94% of reductive *dsrB* genes and 100% of oxidative *dsrB* genes.

The standards for the 16S rRNA gene were obtained from the oil field isolate *Sulfurimonas* sp. strain CVO (72), which was kindly provided by S. Lahme (Newcastle University). Amplicons were obtained using the primer pair 8F/1392R (73), with an initial denaturation at 95°C for 4 min, 30 cycles of denaturation at 95°C for 1 min, annealing at 55°C for 45 s, and extension at 72°C for 1 min, and a final extension at 72°C for 10 min. The standards for *dsrB* genes were obtained from a pure culture of AK-01 (74), which was kindly provided by A. Suárez-Suárez and A. Sherry (Newcastle University). Amplicons were obtained using the primer pair DSR1728fmix/rDSR4rmix, with an initial denaturation at 95°C for 5 min, 30 cycles of denaturation at 95°C for 15 s, annealing at 55°C for 30 s, and extension at 72°C for 20 s, and a final extension at 72°C for 5 min. The amplicon size was verified by agarose gel electrophoresis, and amplicons were purified with a NucleoSpin gel and PCR cleanup kit (Macherey-Nagel, Germany). The concentrations of purified PCR products were quantified with a Qubit double-stranded DNA (dsDNA) assay kit using a Qubit 3.0 fluorometer (Invitrogen, USA). Assuming the mean mass of a base pair equals 1.1 × 10^−21^ g, the standard gene abundance was estimated using equation 2 (67).

(2) $$\text{Standard gene abundance}=\frac{\text{Concentration of purified PCR products}}{\text{Length of the PCR products}\times \text{Mean mass of a base pair}}$$

For both 16S rRNA gene and *dsrB* gene qPCRs, serial dilutions were made and the linear range of quantification was determined as 10^8^ to 10^3^ genes/µl qPCR mixture. The detection limit for each qPCR assay was set to the concentration of the lowest standard dilution whose *C_T_* significantly differed from the *C_T_* of the NTC, i.e., ∼10^3^ genes/µl qPCR mixture. The total microbial abundance and SRM abundance derived from qPCR assays (cells per milliliter homogenized slurry) were log_10_ transformed and then compared using ANOVA and Tukey’s HSD *post hoc* test.

### 16S rRNA gene sequencing and data analyses.

The 16S rRNA gene sequencing of variable region 4 (V4) was carried out by NU-OMICS (Northumbria University), based on the MiSeq procedure described by Schloss and coworkers (75). The primers F515 and R806 (76) were used in the sequencing. According to TestPrime (http://www.arb-silva.de/search/testprime) and the SILVA small subunit (SSU) database release 138 (last accessed in March 2020), this primer pair can cover 93.7% of archaeal and 92.7% of bacterial 16S rRNA gene sequence diversity, allowing one mismatch (77). Briefly, PCR was carried out using 1× Accuprime Pfx supermix, 0.5 µM each primer, and 1 µl of template DNA under the following conditions: 95°C for 2 min, 30 cycles of 95°C for 20 s, 55°C for 15 s, and 72°C for 5 min, and a final extension at 72°C for 10 min. One positive-control and one negative-control sample were included in each 96-well plate and carried through to sequencing. PCR products were normalized using the SequalPrep normalization kit (Invitrogen), as described in the manufacturer’s instructions, and were combined into four pools. Each pool was quantified using fragment sizes determined with a Bioanalyzer (Agilent Technologies) and concentrations determined with a Qubit fluorometer (Thermo Fisher Scientific). Pools were combined in equimolar amounts to create a single multiplexed library and then denatured using 0.2 M NaOH for 5 min, followed by a 2-min incubation at 96°C. The multiplexed library was diluted to a final concentration of 5 pM, supplemented with 15% PhiX, and loaded onto a MiSeq 500-cycle v2 cartridge.

The downstream data processing and analysis were conducted using mothur v1.42.3 software (78). The processing procedure was performed according to the standard procedure (https://mothur.org/wiki/MiSeq_SOP) accessed in February 2020, with minor modifications (77). Briefly, contigs were constructed by overlapping forward and reverse reads based on quality scores and screened for a maximum length of 275 nucleotides. Sequences were uniquified and aligned to the mothur SILVA v132 reference (https://mothur.org/wiki/Silva_reference_files#Release_132). Chimeras were detected and removed using VSEARCH. Taxonomy was preassigned, and only sequences classified as bacterial or archaeal were reserved for the subsequent clustering. OTUs were defined for the complete data sets by clustering the sequences at 97% sequence similarity and assigning a representative sequence to each of the clusters. Taxonomy was assigned to the OTUs. A total of 9,161 sequences were subsampled from each sample and mapped against the representative sequence of each OTU at 97% similarity to estimate the OTU abundance.

The alpha diversity of the microbial communities from each sample was depicted by the Shannon and Berger-Parker indices obtained based on the subsampled libraries. The indices were calculated using the mothur summary.single function and compared using the Kruskal-Wallis test. The subsequent pairwise comparison was conducted using the Wilcoxon rank-sum test, and the false-discovery rate method was chosen for the *P* value adjustment. For the beta diversity, the phylogenic distances among OTUs were computed based on the representative sequences, and the phylogenetic tree was constructed using the relaxed neighbor-joining method (79) via the mothur clear-cut function. The weighted UniFrac distances among samples were calculated and visualized with NMDS using the Phyloseq package in R (80). OTUs were further binned based on the taxonomic affiliations at the order and genus levels. The SIMPER test was performed using the vegan package in R (81) to identify the main taxa contributing to the beta diversity variance among different test and autoclaved groups, followed by the Kruskal-Wallis test to confirm the significance of the variance. The correlations between the NMDS coordinate scores and the relative abundance of the influential taxa were fitted to the NMDS plot using vegan for visualization. Similarly, the correlations between the NMDS coordinate scores and the metavariables, i.e., the concentrations of dissolved sulfide, sulfate, acetate, propionate and butyrate, were also fitted to the NMDS plot. All statistical tests were conducted with R v3.6.2.

### Data availability.

Raw amplicon sequence data (fastq format) generated in this study are available in the NCBI Sequence Read Archive (SRA) (BioProject accession number PRJNA673232).
