# Supplementary material for: Long-Term Biocide Efficacy and Its Effect on a Souring Microbial Community
Source: Appl Environ Microbiol. 2021 Aug 11;87(17):e00842-21. doi: 10.1128/AEM.00842-21 (PMC8357289; doi:10.1128/AEM.00842-21)
Supplement: Supplemental file 1 — Figures S1 to S5. Download AEM.00842-21-s0001.pdf, PDF file, 1.3 MB [file aem.00842-21-s0001.pdf]

Supplementary material

Long-term biocide efficacy and its effect on a souring microbial community

Xiang Shi[a], Daiane A. F. Oliveira[b], Lea Holsten[c], Katrin Steinhauer[c], Julia R. de Rezende[a]*

[a]The Lyell Centre, Heriot-Watt University, Edinburgh, UK

[b]Department of Oceanography, Federal University of Bahia, Salvador, BA, Brazil

[c]Schülke & Mayr GmbH, Norderstedt, Germany

Running Head: Long-term biocide effect on a souring community

Key words: reservoir souring; biocide; oilfield microbiology; sulfate reduction; microbial control; glutaraldehyde

*Address correspondence to Julia R. de Rezende, j.de.rezende@hw.ac.uk - The Lyell Centre, Heriot-Watt University, Research Avenue South, Edinburgh, EH14 4AS, UK

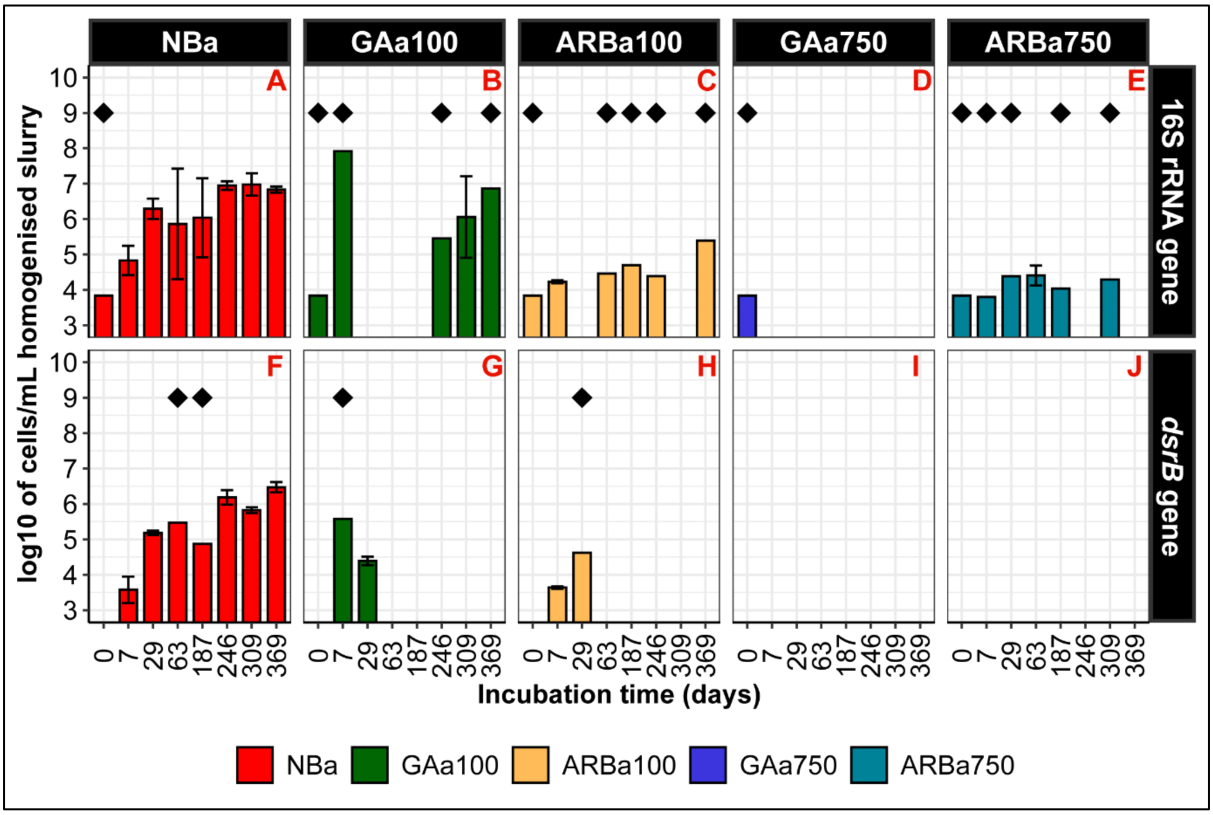

Figure S1. Log$_{10}$ transformed total microbial abundance (top row) and SRM abundance (bottom row) estimated for each autoclaved group based on 16S rRNA gene and dsrB gene qPCR respectively. A, F) NBa; B, G) GAa100; C, H) ARBa100; D, I) GAa750; E, J) ARBa750. For samples with target gene abundance above detection limit, an average of 4.9 copies of 16S rRNA gene/cell for total microbial abundance (https://rrndb.umms.med.umich.edu/, last accessed in May 2019); and an average of 1 copy of dsrB gene/cell for SRM abundance were used to convert the abundance of 16S rRNA genes and dsrB genes to the number of cells respectively (1, 2). The samples with target gene abundance below detection limit were excluded from the subsequent plotting. Values shown are the average among microcosm replicates and the error bars represent the standard deviation among the replicates. N=2 for all bars except for the bars indicated by closed diamonds, for which N=1. The day 0 values in all panels are the same, referring to the microbial abundance obtained for the inoculum. Where data is only available from one replicate (i.e. N=1) for two or more time points, the data is usually from the same replicate

over time, with the exception of panels B and C. In panel B, data is only available from microcosm 10 on day 7 and from microcosm 9 on days 246, 309 and 369. In panel C, data is only available from microcosm 19 on day 7 and from microcosm 20 on days 63, 187, 246 and 369. The average amplification efficiency was $E_{standard}$ =1.68±0.02 and $E_{template}$=1.70±0.05 for 16S rRNA gene qPCR; $E_{standard}$ =1.66±0.01, $E_{template}$=1.71±0.04 for dsrB gene qPCR. The detection limit was ca. 10^3 genes/µL for all 16S rRNA gene and dsrB qPCR assays. To avoid the underestimation or overestimation of the 16S rRNA gene and dsrB gene, the target gene abundance was estimated based on one point correction (OPC) method for every sample (3). Serial dilutions of standards representing 10^4 -10^7 genes/µL were chosen as the OPC references to ensure the C_q of the references falls between 8 and 30.

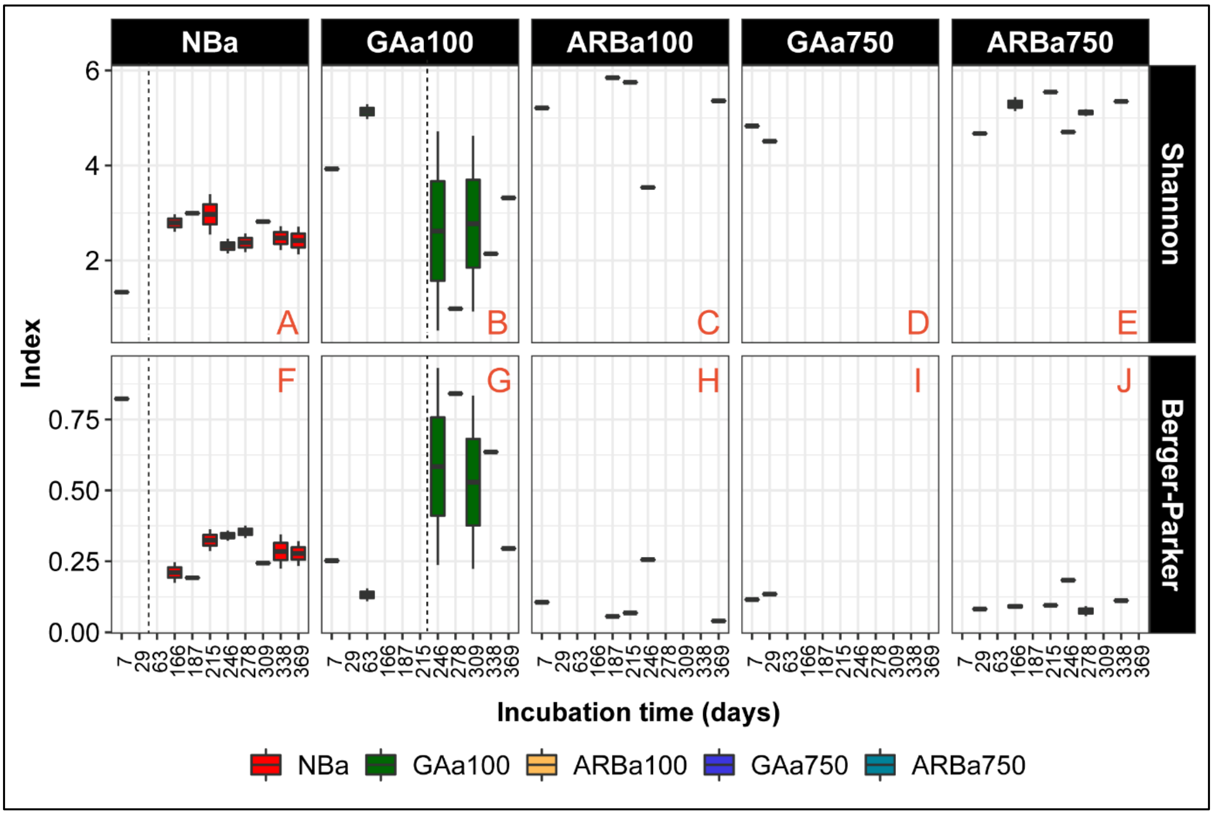

Figure S2. Shannon index (top row) and Berger-Parker index (bottom row) estimated for each autoclaved group based on 16S rRNA gene sequencing. A, F) NBa; B, G) GAa100; C, H) ARBa100; D, I) GAa750; E, J) ARBa750. The vertical dashed lines in panel A and F indicate when souring control started to fail in at least one of the replicates in NBa; the vertical dashed lines in panel B and G indicate when souring control started to fail in GAa100 (microcosm no. 9). Souring control did not fail in the remaining GAa100 replicates, or ARBa100, GAa750 and ARBa750 during the experiment. The days with no data points indicate that data is not available from that day in the corresponding group due to insufficient sequencing reads.

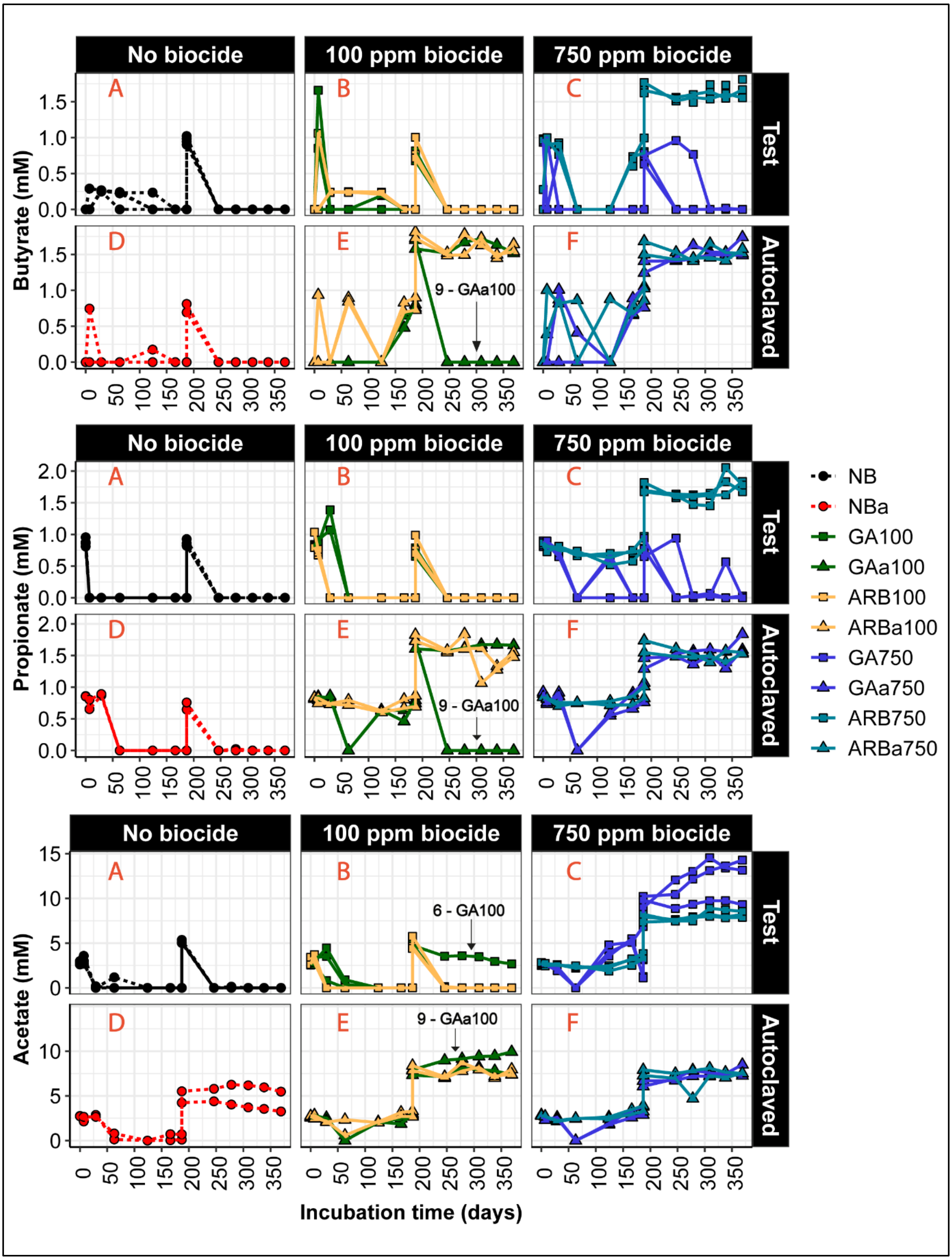

Figure S3. VFA concentration (mM). The top plot shows butyrate concentration, the middle plot shows the propionate concentration and the bottom plot shows the acetate concentration. In each plot, the top row (panels A, B, C) shows test group and the bottom row

(panels D, E, F) shows their respective autoclaved controls. A, D) No-biocide controls; B, E) Microcosms treated with 100 ppm biocide. Green lines and green solid squares indicate GA100. Yellow lines and yellow solid squares indicate ARB100. C, F) Microcosms treated with 750 ppm biocide. Blue lines and blue solid squares indicate GA750. Cyan lines and cyan solid squares indicate ARB750. Replicates referred to specifically in the text are pointed out by arrows, and the format of the arrow label is: “Microcosms No.” – “Group Name”.

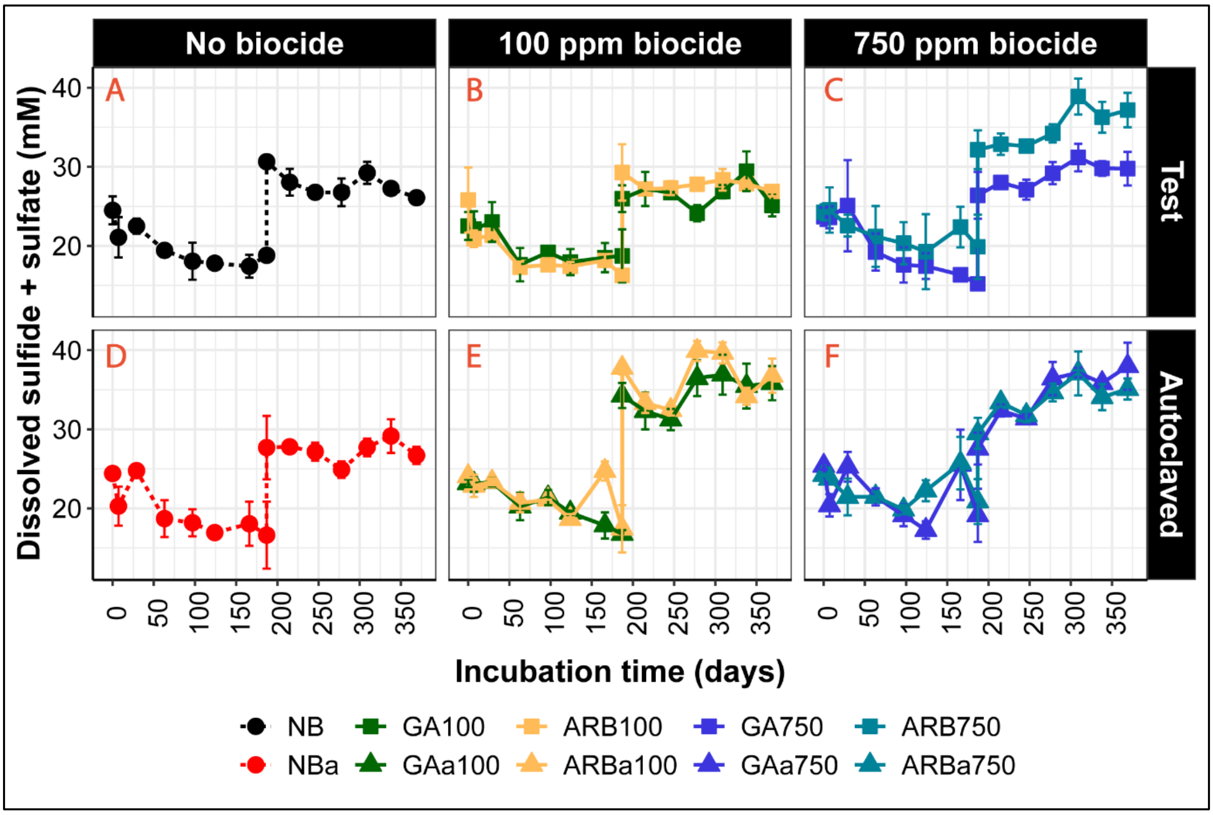

Figure S4. Sum of sulfur concentration (mM). The sum of sulfur concentration equals the sum of the measured dissolved sulfide concentration and the measured sulfate concentration. The top row (panels A, B, C) shows test microcosms and the bottom row (panels D, E, F) shows their respective autoclaved controls. A, D) No-biocide controls; B, E) Microcosms treated with 100 ppm biocide. Green lines and green solid squares indicate GA100. Yellow lines and yellow solid squares indicate ARB100. C, F) Microcosms treated with 750 ppm biocide. Blue lines and blue solid squares indicate GA750. Cyan lines and cyan solid squares indicate ARB100. Results shown were the average results of the replicates (N=3 for A-C. N=2 for D-F). The error bars indicate the standard deviation.

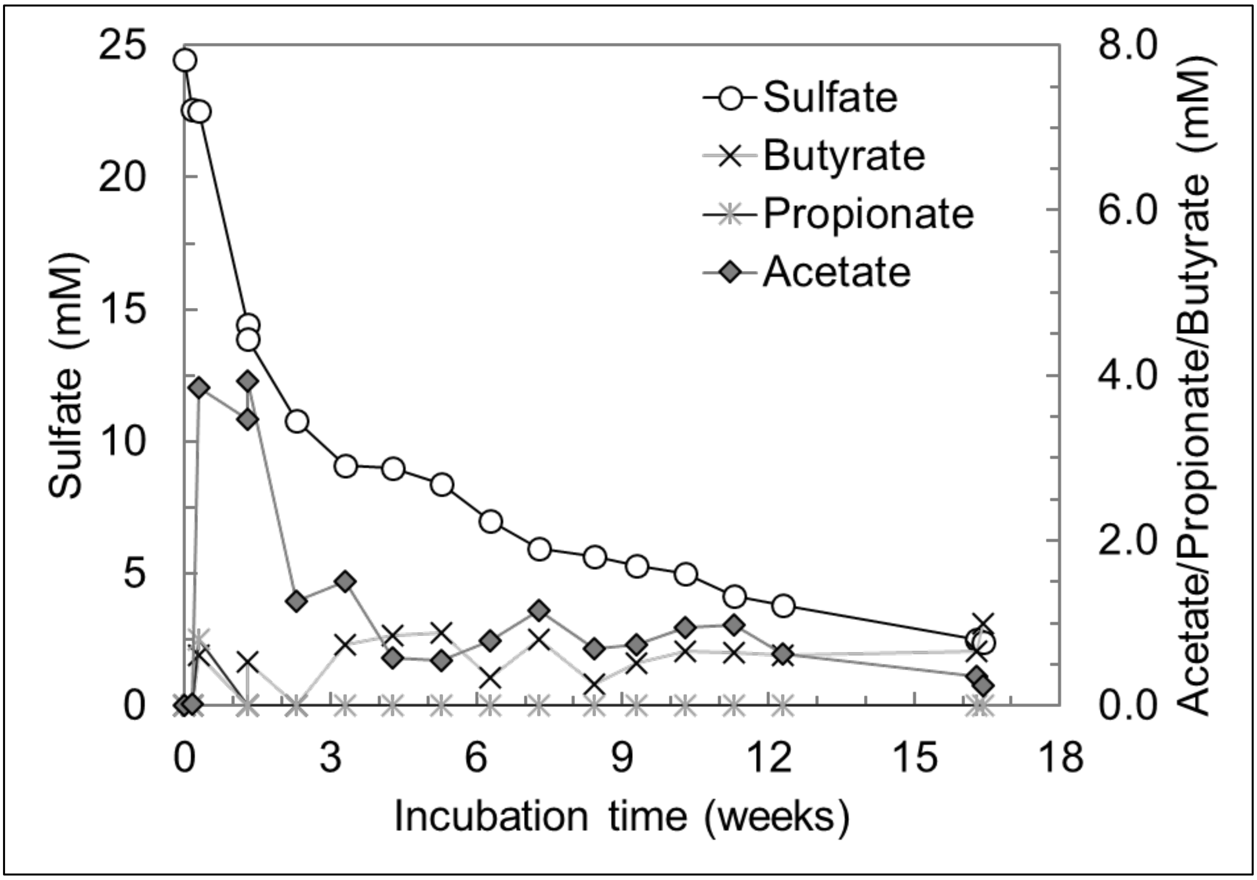

Figure S5. Substrates concentration measured from the experiment inoculum by the end of pre-enrichment.

References

1. Stoddard SF, Smith BJ, Hein R, Roller BRK, Schmidt TM. 2015. rrnDB: improved tools for interpreting rRNA gene abundance in bacteria and archaea and a new foundation for future development. Nucleic Acids Res2014/11/20. 43:D593–D598.

2. Kondo R, Nedwell DB, Purdy KJ, Silva SQ. 2004. Detection and Enumeration of Sulphate-Reducing Bacteria in Estuarine Sediments by Competitive PCR. Geomicrobiol J 21:145–157.

3. Callbeck CM, Sherry A, Hubert CRJ, Gray ND, Voordouw G, Head IM. 2013.

Improving PCR efficiency for accurate quantification of 16S rRNA genes. J Microbiol Methods 93:148–152.
